# Facial mimicry and metacognitive judgments in emotion recognition are distinctly modulated by social anxiety and autistic traits

**DOI:** 10.1038/s41598-023-35773-6

**Published:** 2023-06-15

**Authors:** Julia Folz, Rüya Akdağ, Milica Nikolić, Henk van Steenbergen, Mariska E. Kret

**Affiliations:** 1 Department of Cognitive Psychology, Institute of Psychology, Leiden University, Leiden, The Netherlands; 2 Leiden Institute for Brain and Cognition (LIBC), Leiden University, Leiden, The Netherlands; 3 Research Institute of Child Development and Education, University of Amsterdam, Amsterdam, The Netherlands

**Keywords:** Human behaviour, Biomarkers

## Abstract

Facial mimicry as well as the accurate assessment of one's performance when judging others’ emotional expressions have been suggested to inform successful emotion recognition. Differences in the integration of these two information sources might explain alterations in the perception of others’ emotions in individuals with Social Anxiety Disorder and individuals on the autism spectrum. Using a non-clinical sample (N = 57), we examined the role of social anxiety and autistic traits in the link between facial mimicry, or confidence in one’s performance, and emotion recognition. While participants were presented with videos of spontaneous emotional facial expressions, we measured their facial muscle activity, asked them to label the expressions and indicate their confidence in accurately labelling the expressions. Our results showed that confidence in emotion recognition was lower with higher social anxiety traits even though actual recognition was not related to social anxiety traits. Higher autistic traits, in contrast, were associated with worse recognition, and a weakened link between facial mimicry and performance. Consequently, high social anxiety traits might not affect emotion recognition itself, but the top-down evaluation of own abilities in emotion recognition contexts. High autistic traits, in contrast, may be related to lower integration of sensorimotor simulations, which promote emotion recognition.

## Introduction

The expression “her face says it all” exemplifies the fundamental contribution of nonverbal signals and cues in the communication of inner states^1, 2^. According to social-functional approaches, emotional expressions are crucial in guiding social interactions by informing about others’ states, evoking coordinated emotional responses, and incentivizing social behavior^3, 4^. The accurate identification of an observed emotional expression is a key component in the interpretation of an expresser’s emotional state, yet links between emotion recognition and other processes underlying emotion perception are still not well described. Facial mimicry, that is the mirroring of an observed expression, is one process that has been suggested to promote the recognition of others’ emotions^5, 6^ (see Facial mimicry paragraph for further details). In contrast to this bottom-up information channel, the top-down assessment of one’s recognition performance, a metacognitive process, might also provide relevant feedback about emotion processing^7^ (see Metacognition paragraph for further details). Importantly, alterations in the processing of others’ emotions, as well as in mimicry and metacognition^8, 9^ have been reported for various mental health and neurodevelopmental conditions, such as Social Anxiety Disorder (SAD) and Autism Spectrum Disorder (ASD). Research in both clinical populations has further revealed a link between alterations in processing other's emotional expressions and social interaction difficulties^10, 11^. The current study examines the putative associations between facial mimicry and confidence in emotion recognition abilities (i.e., a metacognitive judgment) with actual emotion recognition performance, as well as their potential alterations associated with social anxiety and autistic traits.

### Mimicry and emotion recognition

When observing an individual expressing an affective state via the face, people tend to automatically mirror the observed facial expression—a phenomenon called facial mimicry^12^. Distinct changes in activity over two muscle regions, the Zygomaticus Major and the Corrugator Supercilii (for simplicity referred to as “zygomaticus” and “corrugator” hereinafter) have been consistently reported in response to videos of emotional displays: Strongest evidence has been found for an increase in zygomaticus activity when happy facial expressions were viewed, together with a decrease in corrugator activity^5, 13–18^. Enhanced corrugator activity, in contrast, has been linked to the perception of anger displays^5, 13, 16–19^ and, less pronounced, for sadness displays^5, 17, 18^.

Importantly, instead of being only an epiphenomenon, facial mimicry has been proposed to aid emotion recognition^5, 20, 21^. In line with seminal theories on emotion^22, 23^, peripheral signals, such as facial expressions, can not only inform the producer about the physiological effects of emotions via interoceptive pathways^24^. “Facial feedback”^6^ might also act as an information source when another individual’s expression is automatically mirrored, serving as a sensorimotor simulation of another person’s emotional state^25^. This view was supported by studies that showed a decline in emotion recognition performance if facial mimicry was voluntarily^26^ or artificially^27^ blocked. Yet, recent meta-analyses suggest that the effects of facial feedback on affective judgments, including emotion recognition, are not consistent^28, 29^. Moreover, facial mimicry does not seem to be a requirement for successful emotion recognition: A study in patients with Möbius syndrome has shown that, despite facial paralysis, these patients could still accurately recognize emotional expressions^30^.

### Metacognition and emotion recognition

Metacognition describes the monitoring of one’s own cognitive processes and has been claimed to be an immanent feature of human social interactions^31^. Nevertheless, it is scarcely researched in the domain of emotion recognition. According to the few available studies on emotion recognition in healthy adults, a reliable metacognitive resolution (i.e., a clear subjective discrimination between correct and incorrect recognition), together with a general overconfidence has been found^32, 33^. Furthermore, only direct trial-by-trial ratings, which can be used to estimate ‘relative meta-accuracy’, and not global beliefs about one’s abilities, were found to be predictive of performance in emotion recognition tasks^7^.Thus, while global metacognitive beliefs seem to be biased, confidence in one’s emotion recognition skills (i.e., a metacognitive judgment) can act as a reliable feedback mechanism in an emotion recognition context.

### Emotion recognition alterations in SAD and ASD

While emotion recognition difficulties have sporadically been reported in SAD^34^, most research did not find deficits^35^ or even found a higher sensitivity, reflected by an emotion detection at lower expression intensities, to emotional expressions^36, 37^. Heightened attention to social cues also stands at the basis of established theoretical models of SAD^38, 39^ and has predominantly received support in form of a “negativity bias”^40–42^. In other words, negative expressions automatically attract more attention and are avoided at the same time, they are integrated more strongly in judging the self in social interactions, they are remembered better, and even ambiguous expressions are more likely to be judged negatively. Correspondingly, not clinically diagnosed individuals with high social anxiety trait levels have shown an emotion recognition advantage^43^, and specifically better recognition of negative expressions^44, 45^. For individuals on the autism spectrum, in contrast, difficulties in visual emotion recognition paradigms in which emotional facial or bodily expressions had to be matched to samples or labelled have mainly been described for all basic emotions, and, particularly, for fear^46–48^ (however, see^49^). Thus, quite specific particularities in facial emotion recognition have been associated with SAD and ASD. Factors that could be linked to, and potentially even contribute to, the occurrence of those particularities are, however, not well described yet.

In past research, individual differences in autistic traits and social anxiety traits have also been related to the usage of different strategies to recognize emotional expressions. When labelling full-body emotional expressions, high compared to low socially anxious individuals have been shown to attend to faces less, and more to expressive hands, thus using different visual cues^50^. In a study comparing recognition of sadness in static facial expressions versus point light displays, only individuals with low autistic traits, compared to individuals with high autistic traits, showed a recognition advantage for sad faces. Fear, in contrast, could be better recognized in point-light-displays by individuals with low autistic traits, and in faces by individuals with high autistic traits^51^. These findings suggest that, depending on clinical trait levels, different features might be used to identify others’ emotional states. Recently, it has even been suggested more broadly that emotions reach awareness via different pathways in individuals on the autism spectrum compared to neurotypical individuals^52^. Differences in processing facial emotional expressions, despite unimpaired emotion matching performance, have already been reported in autistic children on a neural level^53^. In our study with healthy participants, we aimed to explore whether the link between emotion recognition and two processes that have been suggested to promote emotion recognition, namely facial mimicry and metacognitive judgments, differs depending on social anxiety and autistic traits.

### Altered mimicry in emotion recognition in SAD and ASD

Studies investigating the effects of social anxiety (disorder) on facial mimicry have reported inconsistent results: while some studies found intact mimicry in non-clinical but high socially anxious individuals^19, 54^, others demonstrated enhanced mimicry of negative expressions and diminished mimicry of positive ones^55, 56^ or stronger differential muscle activity between happy and angry expressions, for both the zygomaticus and the corrugator^57^. The literature on ASD gives a clearer picture: reduced automatic mimicry in individuals on the autism spectrum has been reported in many studies^8, 58^. Importantly, this reduction could not be explained by a generally lower facial expressiveness or an inability to mimic expressions, but by a mismatch between observed and produced facial muscle activity patterns^59–61^. Only few studies have described differences in facial mimicry alterations between different emotion categories, and findings are inconsistent. Namely, mimicry of angry, but not happy, facial expressions was reduced with higher autistic trait levels in females in one study^62^, while reduced mimicry of happy, but not sad, expressions has been related to higher autistic traits in another study^63^. Whether observed reductions in facial mimicry in high autistic trait levels are also linked to differences in emotion recognition performance has, however, not directly been investigated yet.

### Altered metacognition in emotion recognition in SAD and ASD

Reduced metacognitive abilities have been proposed as a shared characteristic in different psychiatric disorders^9, 64^. Theoretical accounts on the development and maintenance of SAD have highlighted the importance of a negatively biased view on one’s own performance in a social context^38, 39^, together with an excessive monitoring of the self^65^. This global negative judgment of one’s own abilities might have evolved via repeated underestimation of (social) abilities^66^. However, metacognitive abilities in emotion recognition have yet not been directly tested in individuals with SAD. In contrast, the few studies on metacognitive judgments of social cognition in individuals on the autism spectrum have suggested a complex pattern of alteration. Some studies reported no differences between neurotypical individuals and individuals on the autism spectrum in calibrating confidence judgments to emotion recognition performance, that is, higher confidence rating for more accurate or faster recognition^67, 68^. A more recent study, however, found evidence for both an over- and underconfidence in contrast to actual performance in social cognitive tasks, including emotion recognition, in individuals on the autism spectrum compared to neurotypical individuals^69^. Both expressing low confidence in accurate trials as well as high confidence in incorrect trials should be reflected in a reduced metacognitive sensitivity, which Fleming and Lau^70^ defined as “the extent to which confidence discriminates between correct and incorrect trials” (p. 2). Given the limited knowledge about metacognition in the domain of emotion recognition and its relation to SAD and ASD, the current study aimed to explore two assumptions: (1) whether the negatively biased assessment of one’s performance in social situations in people with high social anxiety trait levels also translates to emotion recognition, and (2) whether the decreased metacognitive sensitivity related to higher autistic traits in the social-cognitive domain also specifically holds for emotion recognition performance.

### Objectives of the current study

In the current study, we examined whether social anxiety and autistic traits modulate the links between facial mimicry and emotion recognition as well as between confidence judgments in own emotion recognition skills and emotion recognition. To investigate this, our participants first passively viewed naturalistic video clips of different facial expressions of emotion while we measured their facial muscle activity. In a subsequent task, participants indicated how strongly they associated the expressions with distinct emotion categories and were asked how confident they were in their judgments. Despite sharing social interaction difficulties in the global disorder definitions, previous research on emotion recognition, facial mimicry, and metacognitive judgments showed specific alterations associated with SAD and ASD. Therefore, we also expected to find distinct modulations for the two trait dimensions.

More specifically, confirming the suggested heightened sensitivity to social cues, higher levels of social anxiety were expected to be related to a recognition advantage of facial expressions, resulting in higher accuracy rates. According to the negativity bias findings, this advantage should be specifically pronounced for negative expressions (i.e., anger, fear, and sadness). As individuals with SAD have been found to report a generalized underconfidence in their social skills, we expected that, despite an improved emotion recognition performance, elevated social anxiety traits would be related to reduced overall confidence in the performance. Moreover, based on observations that confidence judgments were predictive of emotion recognition accuracy in healthy subjects, we would like to explore whether the scaling of confidence judgments to actual emotion recognition performance is altered depending on social anxiety traits. In line with a proposed facilitating role of facial mimicry in emotion recognition, stronger facial mimicry might be assumed with higher social anxiety traits. Empirical evidence for both relationships, social anxiety (disorder) and facial mimicry as well as facial mimicry and emotion recognition, is, however, inconclusive. Therefore, we aimed to directly test whether the role of facial mimicry in emotion recognition is altered depending on an individual’s social anxiety traits.

Regarding autistic traits, we expected to observe an overall worse recognition of naturalistic dynamic facial expressions in association with higher levels of autistic traits, which should be most pronounced for fearful faces. Higher autistic trait levels were further expected to be associated with less facial mimicry, and this reduction was expected to be strongest for negative expressions. Furthermore, as automatic facial mimicry has been suggested to be impaired in ASD, the information about facial muscle activity might also be less well integrated in emotion recognition. Accordingly, we explored whether a positive relationship between facial mimicry and emotion recognition would be less pronounced in individuals with higher autistic trait levels. Lastly, extending on the few findings in clinical samples, we expected lower metacognitive sensitivity in relation to higher autistic traits. Hence, confidence judgments should be less predictive of actual emotion recognition accuracy in individuals with higher autistic trait levels. Given the little and inconclusive evidence on metacognition in emotion recognition in healthy and clinical populations, our analyses regarding this research question were explorative.

## Methods

### Participants

Fifty-seven healthy participants were recruited from the Leiden University student population (50 female and seven male). Their ages ranged from 18 to 30 years old (*M* = 22.75, *SD* = 3.27) and they all reported normal or corrected-to-normal vision. None of the participants reported current or past psychological or neurological disorders. Participation in the study was voluntary and written consent was obtained prior to the experiment. Participants received either two university credits or a monetary reward of six euros as reimbursement. The study has been executed in accordance with the Declaration of Helsinki and approved by the local ethics committee of the Faculty of Social and Behavioral Sciences at Leiden University (# 2020-02-10-M.E. Kret-V1-2117).

In the scope of a Master thesis project, an a priori power analysis was run for this study, treating clinical traits as a categorical variable (low vs. high trait levels). Based on a similar previous study that found significant group effects with medium effect sizes^71^, we estimated our ideal sample size with the Power Analysis for General ANOVA application (PANGEA)^72^. With 30 participants per group, hence 60 participants in total, a group effect of d = 0.50 should be found with a power of 0.901. Because of the COVID-19 regulations in the Netherlands, we had to stop data collection prematurely and ended up with 57 participants in total (56 participants: power of 0.879). For the analyses in this manuscript, we treated the clinical trait dimensions as continuous variables, thereby increasing the validity of the approach as well as statistical power^73^.

### Stimuli

**Figure 1:**
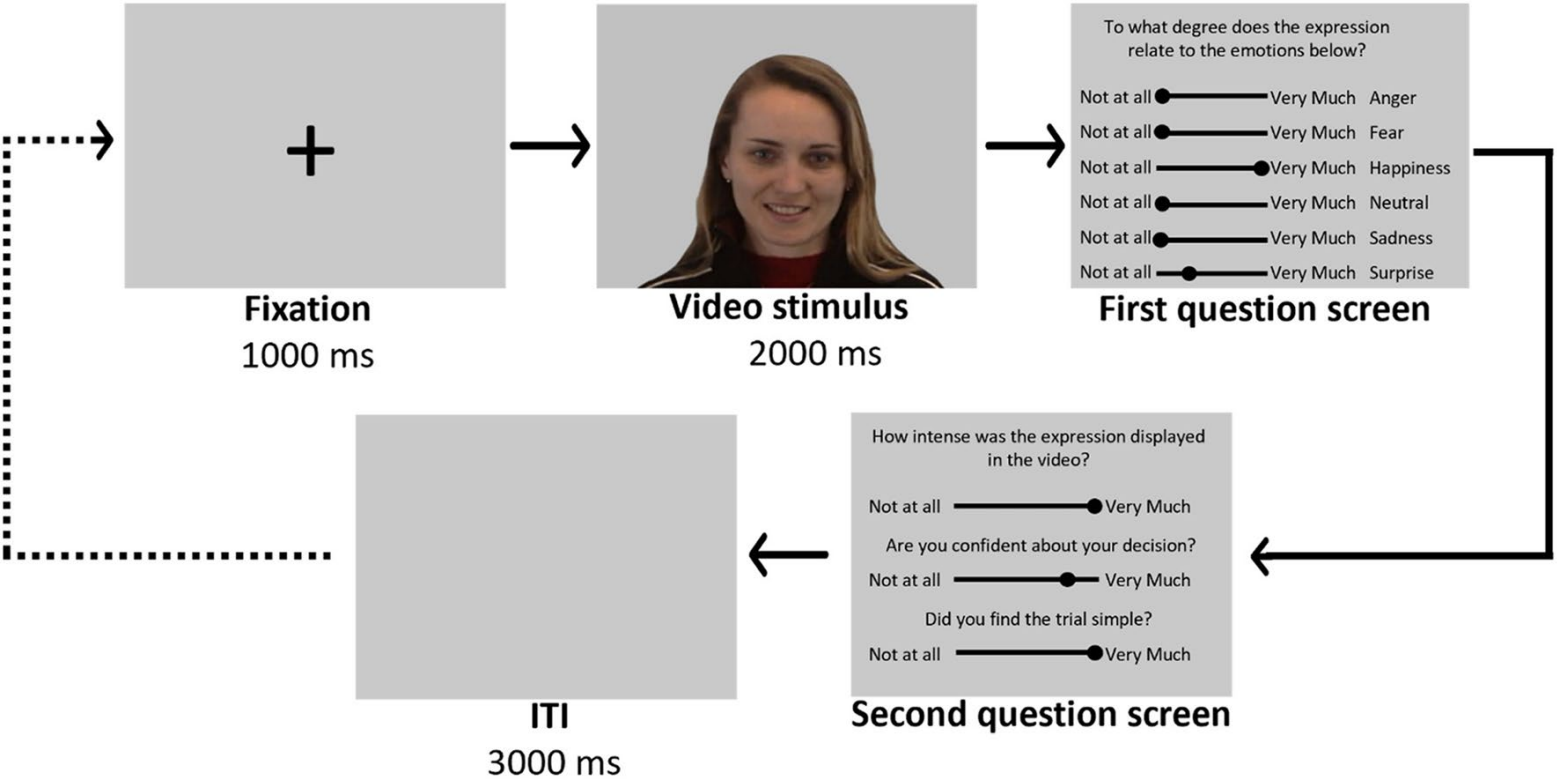
Illustration of a Facial Emotion Recognition task trial. Each trial started with a fixation screen and ended with an intertrial interval (ITI) screen. The dotted line indicates that this sequence was repeated until all 60 videos were presented once.

Following the call for more naturalistic stimuli in research on emotion perception, we chose the FEEDTUM database^74^ as a source for our stimuli. This database encompasses videotaped spontaneous (i.e., non-instructed) reactions to video clips inducing the six different basic emotions and neutral control expressions. All depicted individuals provided informed consent for the usage of the videos for research purposes, including distribution and publication of the material, in the original study. Permission to use the material under CC-by and to publish example images in scientific journals, such as in Fig. 1, was granted to the first author of this study by the creators of the database. Based on the choice of stimuli in a previous study investigating facial mimicry and emotion recognition in depression^71^, we included facial expressions of anger, fear, happiness, sadness, surprise, and neutral. Disgust was not included, which is a basic emotion that (next to surprise) is typically less investigated in studies on emotion recognition alterations in SAD and ASD^35, 46^. For each facial expression, video clips of ten individuals (five females and five males) were selected based on the following decision pipeline: first, videos were judged on their quality, and blurry or shaky videos were excluded. Second, individuals wearing glasses or individuals with hair in front of their eyes were excluded as these features made their facial expressions more difficult to recognize. Lastly, all remaining video clips were evaluated by the automated facial expression recognition software FaceReader 7.1^75^ to ensure that the emotion label of the stimulus provided by the database could also be detected in the video. After this selection procedure, the video clips were cut to a uniform length of 2 s (500 ms neutral expression followed by 1500 ms of each category’s expression). Lastly, the video clips were standardized by removing the original backgrounds using Adobe After Effects^76^, and by replacing them with a uniform gray colored background (RGB color code: 145, 145, 145). This led to a total of 60 two-second videos with a grey background showing ten individuals (five males and five females) per facial expression.

### Procedure

Participants were brought to a quiet room, in which they were given written and verbal instructions about the experimental procedure. After filling in the informed consent form, electrodes were attached to the participants’ faces for the facial electromyography recordings (see Measurements section). During the tasks, participants were seated in 50 cm distance of a Philips screen with a resolution of 1920 × 1080 pixels (23.6"), on which the stimuli (720 × 480 pixels, average visual angle: 22.12° horizontal and 14.85° vertical) were presented using E-Prime 3.0 software^77^. The grey background colour of all screens was set to the background colour of the stimuli (RGB color code: 145, 145, 145). The same 60 emotional facial expression videos were presented in a random order in two consecutive tasks, a passive viewing task during which the participants’ facial muscle activity was recorded, and a facial emotion recognition task. The rationale behind the two separate tasks was to avoid that participants would be biased in their perception and their facial mimicry responses in the passive viewing task by being aware of the possible emotion category labels (i.e., top-down modulation). In the passive viewing task, participants were instructed to only look at the stimuli without performing any action. Each trial started with the presentation of a black fixation cross against a grey background for one second, and was followed by one of the 60 video stimuli for two seconds. The end of a trial was marked by a grey inter-trial interval (ITI) screen, which appeared with a jittered duration of either 5750, 6000, or 6250 ms. To account for the possibility of missing observations due to noisy data, participants viewed each of the 60 videos twice, in two separate blocks, resulting in 120 trials in total. Between the blocks, participant could take a self-paced break. The passive viewing task lasted around 20 min in total. After the passive viewing task, the electrodes were detached from the participants’ faces and the experiment continued with the facial emotion recognition task. In this second task, the participants viewed all 60 video stimuli once again (thus three times in total) but were now instructed to answer questions about them. Similar to the passive viewing task, each trial started with the fixation cross screen for one second and one of the 60 video stimuli (2 s) was presented afterwards. Once the video disappeared, participants were asked to judge the displayed expression. More specifically, on the first question screen, they were asked to rate the expression according to its representativeness of the six expression categories that could be displayed: anger, fear, happiness, neutral, sadness, and surprise ('To what degree does the expression relate to the emotions below?'). Each expression category was accompanied by horizontal sliders ranging from 'not at all' to 'very much' and participants had to move the slider to indicate their judgment. The values of the sliders ranged from 0 to 100 in steps of 10 (0, 10, 20, etc.), which were not visible to the participants. The next screen contained three questions: (1) 'How intense was the expression displayed in the video?' to measure perceived emotional intensity; (2) 'Are you confident about your decision?' to measure confidence in own performance; and (3) 'Did you find the trial simple?' to measure simplicity^71^. Again, all questions were accompanied by a slider ranging from 'not at all' to 'very much' with underlying values ranging from 0 to 100 in steps of 10. Thus, higher scores indicated higher ratings on perceived intensity, confidence, and simplicity, respectively. After the second question screen, a grey inter-trial interval (ITI) screen appeared for three seconds. In total, the participants completed 60 trials, rating all stimuli from the first task, plus three additional practice trials showing a different individual to familiarize them with the task. After 30 trials, participants could take a self-paced break and the entire facial emotion recognition task lasted approximately 25–30 min. A visualization of one facial emotion recognition trial can be found in Fig. 1.

Importantly, only the ratings on the association of the displayed expression with the expression categories (first question screen) and the confidence ratings on the second screen were relevant to answer our hypotheses. As we did not formulate specific hypotheses about alterations in perceived emotional intensity ratings related to social anxiety or autistic traits, explorative analyses on this rating variable can be found in the Supplemental Materials S1. Furthermore, the simplicity ratings provided insights in how difficult emotion recognition with this novel stimulus set was perceived. Overall, happy expressions received the highest simplicity ratings (*M* = 69.39, *SD* = 27.15), followed by surprised expressions, (*M* = 62.51, *SD* = 25.35), fearful expressions (*M* = 50.93, *SD* = 26.19), angry expressions (*M* = 45.05, *SD* = 25.66), neutral expressions (*M* = 36.42, *SD* = 33.74), and sad expressions (*M* = 36.19, *SD* = 25.56). Some might consider differences in simplicity between emotion categories as potential confound in predicting emotion recognition accuracy (i.e., higher simplicity ratings might systematically be linked to more accurate choices). Yet, these differences have been proposed to arise due to factors that are inherently linked to the specific emotional expression, such as a higher familiarity with increased exposure in daily life^78, 79^. We aimed for higher ecological validity in examining facial emotion recognition by using spontaneous and non-acted expressions in the current study. Controlling for simplicity, in contrast, would detach our results from emotion recognition in everyday life, which is why we decided against it. Lastly, participants filled in the Liebowitz Social Anxiety Scale (LSAS)^80^ and the Autism Spectrum Quotient (AQ; see Measurements section)^81^. Upon completion of the experiment, participants were given a written and verbal debriefing about the goal of the study and were reimbursed. In total, the experiment lasted around 55 min, including instructions and the attachment of the facial electromyography electrodes.

### Measurements

#### Facial electromyography (fEMG)

Facial electromyography (fEMG) was measured on the left side of the face of all our participants, following the guidelines of Fridlund and Cacioppo^82^. To specify, two reusable 4 mm Ag/AgCl surface electrodes were placed over the Corrugator Supercilii region, which allowed us to measure mimicry responses to sad, fearful, and angry expressions (according to the EMFACS definition) as well as to happiness expressions (as shown in previous research). Other two electrodes were placed over the Zygomaticus Major region, which allowed us to measure mimicry responses to happy expressions. Additionally, a ground electrode was added on the top of the forehead. The signal was transmitted and amplified using a Biopac MP150 system combined with a BioNomadix 2 channel wireless EMG amplifier. Data recordings were made in AcqKnowledge 4.3^83^ using a sampling rate of 1000 Hz. Event markers as defined in the E-Prime^77^ tasks were sent via a parallel port and saved within an event marker channel. For data preprocessing, the EMG recordings were loaded into the PhysioData Toolbox^84^ in which they were rectified and filtered with a 28 Hz high cut-off filter, a 500 Hz low cut-off filter, and a 50 Hz notch filter. For each trial, separate epochs were defined for the fixation period (1 s), the first 500 ms of stimulus presentation in which a neutral expression was shown (later defined as *baseline*), the subsequent 1500 ms in which the emotional expression was shown (later defined as *response*), and the first 1500 ms of the blank screen after stimulus presentation. Within these epochs, the EMG signal was downsampled by calculating the average signal within consecutive 100 ms time bins. The combined data from all subjects was then exported into MATLAB for further preprocessing. First, an automated artifact detection, which was inspired by Dignath et al.^85^, was conducted. More specifically, for each subject and each muscle region, we checked the distribution of the EMG signal for extreme values (± 3.5 SDs) in the time bins regarding the absolute value of each time bin and the relative differences between subsequent bins. This was performed in relation to (1) the entire time interval of interest per trial (5 baseline time bins and 15 response time bins) and (2) the distribution of baseline time bins in the same position across trials. If more than 50% of all time bins (20) or more than 50% of the baseline time bins (5) belonging to one trial had extreme values, this trial was entirely excluded from the analysis. Otherwise, the respective time bins were replaced with missing values. Across all subjects, 17 trials (0.002%) were excluded from the corrugator analysis and 150 trials (0.02%) from the zygomaticus analysis. After excluding the marked time bins, a baseline correction was performed by subtracting the mean EMG activity of all baseline time bins belonging to one trial from the respective response time bins. The baseline-corrected EMG data was then z-scored for each participant and each muscle region. Furthermore, the data was summarized on a trial level by averaging the last second of each trial’s response window (last 10 time bins) for each participant and each muscle region as well as by averaging across the two presentations of each of the 60 stimuli to end up with the same amount of observations as for the rating data (trial-averaged data; used as predictor in generalized linear mixed models). Lastly, the data was also summarized on a participant level by creating the average of the same time window across trials for each participant, each muscle region and each emotion category (category-averaged, used as outcome variable in linear models).

#### Questionnaires

##### Social anxiety traits measure

We used the Liebowitz Social Anxiety Scale (LSAS)^80^ to measure self-reported social anxiety traits in our non-clinical sample. The LSAS is designed to assess fear and avoidance levels of individuals with social phobia in a range of social interaction and performance scenarios. The questionnaire contains 24 items in total. Respondents score the items for fear and avoidance separately on a 4-point Likert scale, fear: 0 (= None), 1 (= Mild), 2 (= Moderate), 3 (= Severe); avoidance: 0 (= Never), 1 (= Occasionally), 2 (= Often), 3 (= Usually). The scores are all added up to a total sum of all subscales, with higher scores indicating a higher severity of social anxiety symptoms. One participant had missing data for one questionnaire item and another participant had missing data for two questionnaire items, which were estimated using the mice-package^86^ for multiple imputation. The LSAS showed an excellent internal consistency in our sample (α = 0.91, 95% CI [0.88, 0.95]). LSAS scores ranged from 7 to 73 (*M* = 38.53, *SD* = 17.53), with 30 participants (52.63%) exceeding a score of 30. This score has been described as the best cut-off to discriminate between non-anxious individuals and individuals with SAD^87, 88^. Thus, a broad spectrum of social anxiety trait levels was covered in our sample, with half of the participants showing an indication of clinically relevant social anxiety. The average LSAS scores were considerably higher compared to the healthy validation sample of the LSAS self-report version (*M* = 13.49, *SD* = 23.70)^89^. With a skewness of 0.21 and a kurtosis of 2.08, the distribution of the LSAS scores showed to be slightly platykurtic, yet close to normal (see Fig. S1A in the Supplemental Material).

##### Autistic traits measure

The Autism-Spectrum Quotient (AQ)^81^ is a self-report questionnaire, which was created to measure traits associated with the autism spectrum. The AQ consists of 50 items in total and can be divided into five subscales (10 items each) assessing different domains: social skill, attention switching, attention to detail, communication, and imagination. Respondents indicate how strongly each item applies to them based on a 4-point Likert scale ranging from 1 (= definitely agree), 2 (= slightly agree), 3 (= slightly disagree), and 4 (definitely disagree), and some items are reversely scored to prevent response biases. All item scores are added up to a total sum score, with higher scores reflecting higher autistic trait levels. One participant did not complete the AQ and was therefore excluded from all analyses investigating effects of autistic traits. Furthermore, we had to estimate three single item scores using the mice-package^86^ for multiple imputation as one participant did not respond to one item and another participant did not respond to two items. Internal consistency of the AQ in our sample was good, α = 0.83, 95% CI [0.76, 0.89]. The range of AQ scores was between 2 – 39 (*M* = 16.38, *SD* = 7.34), which is highly similar to meta-analytic results on AQ scores in general population samples (*M* = 16.94, 95% CI [11.6, 20.0])^90^. Only 3 participants (5.26%) had a higher AQ score than 32, which indicates autistic trait levels of clinical significance. The skewness and kurtosis of the AQ score distribution were 1.05 and 4.17 respectively, thus showing a positive skew (see Fig. S1B in the Supplemental Material).

### Data analysis

Spearman's rank correlation revealed that autistic traits and social anxiety traits, reflected by the scores on the two questionnaires, were not significantly associated with each other, *r*_*s*_ = 0.04, *p* = 0.784. Our sample showed both variability within each trait dimension that was similar to studies with larger samples (see Questionnaire section) and independence between the trait dimensions, allowing for separate analyses for the two trait dimensions. Emotion recognition accuracy was calculated by determining the expression category with the highest slider score and comparing it to the predefined category of the stimulus for each trial^71^. If there was a match between the presented and the perceived expression category, a trial was scored as correct (1) whereas it was scored as incorrect (0) in case of a mismatch. Trials in which two expression categories received the same slider scores were discounted from the analysis. To check the robustness of this approach, we re-ran all analyses on accuracy with a relative accuracy score, which was calculated by subtracting the mean score of all other expression categories from score of the correct expression category^91^. The results were overall highly similar and are reported in the Supplemental Materials S1. All analyses were performed in R 4.0.1^92^, using the lmerTest package^93^ for fitting the (generalized) linear mixed models ([G]LMMs), the multcomp package^94^ for general hypotheses testing, the sjPlot package^95^ for creating the tables and both the sjPlot package and ggplot2^96^ for creating the plots.

#### Behavioural analysis

##### Accuracy and confidence

In order to test whether social anxiety traits were associated with better emotion recognition performance for negative expressions and whether emotion recognition accuracy was generally reduced with higher autistic traits, we calculated two binomial GLMMs on accuracy with emotion category (anger, fear, happiness, sadness, surprise, and neutral), the respective trait dimension (autistic traits or social anxiety traits), and their interaction as fixed effects. Both participant ID and the identity of the stimulus were added as random effects (random intercept). Furthermore, we fitted two LMMs on emotion recognition confidence to test the association between confidence level and (i) social anxiety traits, (ii) autistic traits. The models had the same fixed and random effects structure as the accuracy models. Coefficients for the emotion categories (main effects and interactions) were calculated by contrasting each single category against the mean of all categories (sum coding) to determine significant deviations from mean accuracy (main effect) or the mean effect of a trait dimension (interaction). For the neutral category, coefficients were calculated and tested (z-tests) using general hypotheses testing. Lastly, we explored whether the relation between confidence and emotion recognition was altered depending on an individual's clinical trait levels as well as the presented expression. To do so, we added emotion recognition confidence and all 2-way interactions as well as the 3-way interactions with emotion category and the respective clinical trait dimension to the accuracy models. The resulting model fits were the following:

1. LSAS * EMOTION CATEGORY—> EMOTION RECOGNITION ACCURACY
2. AQ * EMOTION CATEGORY—> EMOTION RECOGNITION ACCURACY
3. LSAS * EMOTION CATEGORY—> EMOTION RECOGNTION CONFIDENCE
4. AQ * EMOTION CATEGORY—> EMOTION RECOGNTION CONFIDENCE
5. LSAS * EMOTION CATEGORY * CONFIDENCE—> EMOTION RECOGNITION ACCURACY (exploratory)
6. AQ * EMOTION CATEGORY * CONFIDENCE—> EMOTION RECOGNITION ACCURACY (exploratory)

##### Metacognitive sensitivity

To examine how well an individual's confidence ratings could distinguish between accurate and inaccurate trials in the emotion recognition task, we calculated the hit and false alarm rate pairs with increasing confidence levels (11, according to points on the Likert scale) for each subject and employed the area under the type 2 ROC curve (AUROC2) approach according to Fleming and Lau^70^. More specifically, each confidence level was taken as a criterion to distinguish between low and high confidence trials; starting with a criterion in which only zeroes were regarded as low confidence ratings and all higher values were regarded as high confidence ratings, up until a criterion in which all trials below the highest confidence rating (100) were regarded as low confidence trials and only the highest rating was regarded as high confidence. The resulting probabilities for hits, p(high confidence|correct), and false alarms, p(high confidence|incorrect), were plotted against each other for each confidence level. The resulting area under this ROC2 curve was taken as an index for the subject's metacognitive sensitivity, describing how well an individual's confidence ratings were scaled to actual emotion recognition accuracy. The link to each clinical trait was then tested with a correlational analysis.

#### Facial EMG analysis

##### Facial muscle activity (mimicry)

By measuring facial muscle activity over the Corrugator Supercilii and Zygomaticus major regions, we could assess mimicry responses to angry, happy, sad and fearful expressions, with neutral expressions acting as a reference category. In order to examine whether social anxiety traits are associated to an enhanced mimicry of specifically angry (negative) expressions, we fitted a linear model on the category-averaged corrugator activity (i.e., taking the mean corrugator activity of all trials belonging to the same emotion category) with emotion category, social anxiety traits and their interaction as fixed effects. We also aimed to explore zygomaticus activity for mimicry of happy expressions and, therefore, used the same independent variables to predict category-averaged zygomaticus activity (i.e., taking the mean zygomaticus activity of all trials belonging to the same emotion category). By replacing social anxiety traits with autistic traits in the other two linear models on category-averaged corrugator and zygomaticus activity, we then tested whether typical mimicry patterns are indeed reduced with higher autistic traits (i.e., less corrugator activity for negative expressions (specifically anger), less zygomaticus activity for happy expressions and less decrease in corrugator activity for happy expressions). Coefficients for the emotion categories (main effects and interactions) were calculated by contrasting the respective category against the neutral reference category. Since the residual plots of the two model fits on zygomaticus activity showed violation of the normality assumption as well as heterogeneous error estimates, we calculated non-parametric estimates of the predictor effects and confidence intervals, using 1000 bootstrap iterations, for these two models. As neither main effects of clinical traits nor interaction effects with emotion category were found, the results, including significant effects of emotion category on EMG activity, are reported in the Supplemental Materials (Tables S7–S10).

##### Link between facial muscle activity (mimicry) and emotion recognition accuracy

As outlined in the introduction (see Mimicry and Emotion Recognition section), the association between facial muscle activity and emotions, including mimicry of expressions, is specific for each emotion category. Emotion recognition should only be informed by facial muscle activity that is in line with the assumed mimicry responses for the specific emotion (e.g., increase in zygomaticus activity and decrease in corrugator activity for happy expressions, and increase in corrugator for sad expressions). Therefore, we ran separate analyses for the emotion categories anger, happiness, sadness, and fear to investigate the relationship between intraindividual differences in facial muscle activity and emotion recognition accuracy in the context of varying clinical trait levels. Binomial GLMMs were fitted on emotion recognition accuracy with the trial-averaged EMG activity over the two muscle regions (corrugator and zygomaticus) as distinct predictors, as well as one of the clinical trait scores and both two-way interactions (8 models in total). Similar to the behavioural accuracy models, random intercepts were added for the subject as well as the stimulus identity. This resulted in the following models:

1. LSAS * CORRUGATOR—> ANGER RECOGNITION ACCURACY
2. LSAS * CORRUGATOR + LSAS * ZYGOMATICUS—> HAPPINESS RECOGNITION ACCURACY
3. LSAS * CORRUGATOR—> SADNESS RECOGNITION ACCURACY
4. LSAS * CORRUGATOR—> FEAR RECOGNITION ACCURACY
5. AQ * CORRUGATOR—> ANGER RECOGNITION ACCURACY
6. AQ * CORRUGATOR + AQ * ZYGOMATICUS—> HAPPINESS RECOGNITION ACCURACY
7. AQ * CORRUGATOR—> SADNESS RECOGNITION ACCURACY
8. AQ * CORRUGATOR—> FEAR RECOGNITION ACCURACY

### Ethical Approval

The study was reviewed and approved by the Psychology Ethics Committee of Leiden University (2020-02-10-M.E. Kret-V1-2117).

### Informed consent

Written consent was obtained from all participants.

## Results

### Behavioural results

#### Accuracy in emotion recognition

##### Social anxiety traits

**Figure 2:**
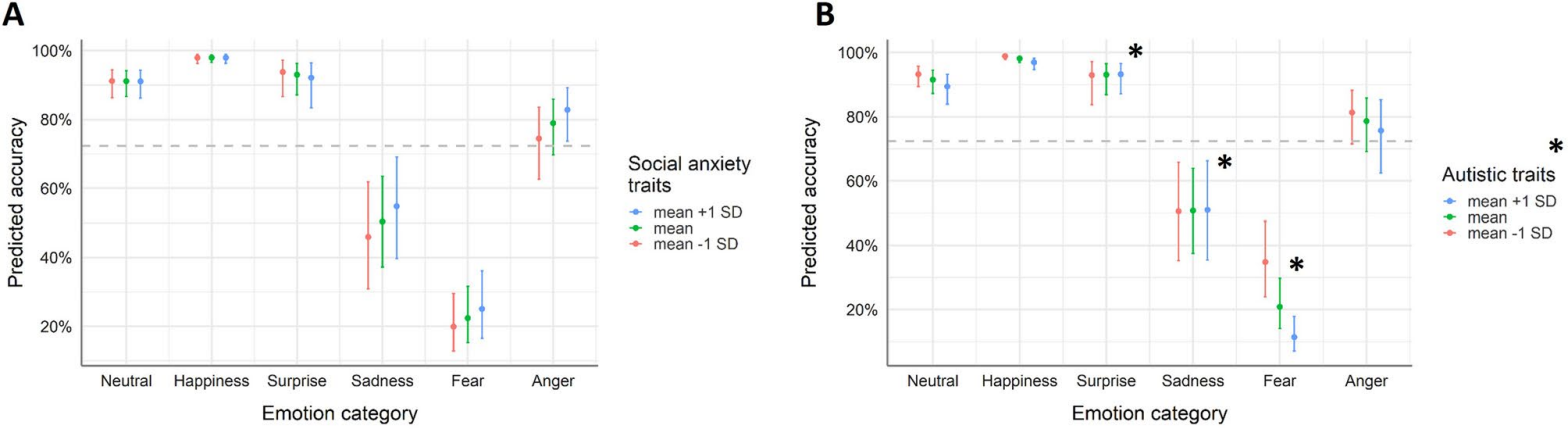
Predicted emotion recognition accuracies depending on (**A**) social anxiety trait levels and (**B**) autistic trait levels by emotion category (anger, fear, sadness, surprise happiness, neutral). In the model fits, accuracy was coded binomial (0–1 values). For illustrative purposes, predicted accuracies for mean values as well as mean values ± 1 SD of the continuous variables social anxiety traits and autistic traits are depicted in percentages. Whiskers represent confidence intervals and significant effects are marked with an asterisk. The dashed horizontal line indicates mean predicted accuracy (across all categories and trait levels).

The first binomial GLMM on emotion recognition accuracy included emotion category, social anxiety traits, and their interaction as predictors. Results showed a significant main effect of emotion category, χ^2^(5) = 702.880, *p* < 0.001. Emotion recognition performance for happy, surprised, and neutral expressions was significantly better than average recognition performance, happy: *OR* = 10.834, *z* = 10.797, *p* < 0.001, surprise: *OR* = 3.027, *z* = 7.654, *p* < 0.001, neutral: *OR* = 2.337, *z* = 6.420, *p* < 0.001. In contrast, sad and fearful expressions were significantly worse recognized than average, *OR* = 0.232, *z* = − 14.336, *p* < 0.001, and *OR* = 0.066, *z* = − 23.445, *p* < 0.001 respectively. All the other effects or interactions were not significant. This suggests that recognition accuracy was predicted by the emotional content displayed in the video, independent of the level of social anxiety traits (see Fig. 2A). An overview of the model fit can be found in the Supplemental Material (see Table S1).

##### Autistic traits

The second binomial GLMM on emotion recognition accuracy included emotion category, autistic traits, and their interaction as predictors. Results showed a significant effect of emotion category, χ^2^(5) = 666.374, *p* < 0.001, a significant effect of autistic traits, χ^2^(1) = 8.985, *p* = 0.003, and a significant interaction between autistic traits and emotion category, χ^2^ (5) = 21.606, *p* = 0.001. The overall negative association between autistic traits and emotion recognition accuracy, *OR* = 0.763, *z* = − 2.998, *p* = 0.003, was most pronounced for fearful expressions, *OR* = 0.637, *z* = − 3.292, *p* = 0.001. Recognition of sad expressions was less negatively affected by autistic traits compared to the overall performance, *OR* = 1.322, *z* = 2.821, *p* = 0.005, and similarly recognition of surprised expressions, *OR* = 1.314, *z* = 2.067, *p* = 0.039 (for all other effects and more detailed information, see Fig. 2B and Table S2 in the Supplemental Material). Thus, the expected overall negative association between autistic traits and emotion recognition performance seems specifically pronounced for fearful facial expressions.

#### Confidence in emotion recognition

##### Social anxiety traits

**Figure 3:**
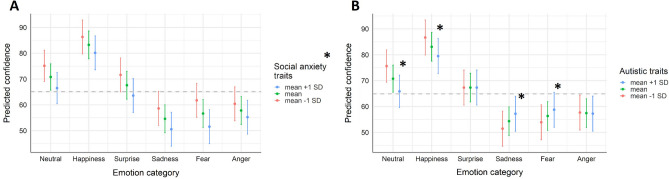
Predicted confidence in emotion recognition depending on (**A**) social anxiety trait levels and (**B**) autistic trait levels by emotion category (anger, fear, sadness, surprise happiness, neutral). For illustrative purposes, predicted accuracies for mean values as well as mean values ± 1 SD of the continuous variables social anxiety traits and autistic traits are depicted. Whiskers represent confidence intervals and significant effects are marked with an asterisk. The dashed horizontal line indicates mean predicted confidence (across all categories and trait levels).

In the first LMM on confidence in emotion recognition, with emotion category, social anxiety traits, and their interaction as predictors, significant effects of both emotion category, *F*(5, 3344) = 118.666, *p* < 0.001, and social anxiety, *F(*1,3344) = 5.362, *p* = 0.024, could be observed. Compared to the average confidence judgments across emotion categories, participants were significantly more confident in judging happy expressions, β = 0.614, *t*(3344) = 19.695, *p* < 0.001, neutral expressions, β = 0.193, *z* = 6.186,* p* < 0.001, and surprised expressions, β = 0.084, *t*(3344) = 2.699, *p* = 0.007. For the other emotional expressions, confidence was significantly reduced compared to the average, angry: β = − 0.247, *t*(3344) =  − 7.932, *p* < 0.001, fearful: β = − 0.287, *t*(6761) =  − 9.209, < 0.001, sad: β = − 0.356, *t*(3344) =  − 11.438, *p* < 0.001. The association between social anxiety traits and confidence was negative, β = − 0.132,* t*(3344) =  − 2.316, *p* = 0.024, and did not vary by emotion category (i.e., no interaction). Thus, independent of the displayed expression, confidence judgments were significantly lower with higher social anxiety trait levels (for a detailed description of the model fit, see Fig. 3A and Table S3 in the Supplemental Material).

##### Autistic traits

In the LMM that included autistic traits instead of social anxiety traits to predict confidence in emotion recognition, the effect of emotion category was significant, *F(*5, 3285) = 118.164, *p* < 0.001, as was the interaction between emotion category and autistic traits, *F(*5, 3285) = 9.531, *p* < 0.001. While for neutral and happy facial expressions confidence was significantly reduced with higher autistic traits compared to the average effect of autistic traits on confidence ratings, β = − 0.148, *z* = − 4.663, *p* < 0.001, and β = − 0.104, *t(*3285) =  − 3.271, *p* = 0.001 respectively, this effect was reversed for displays of fear and sadness. For those two categories, autistic traits were associated with higher confidence ratings compared to the average effect of autistic traits on confidence, fear: β = 0.102, *t(*3285) = 3.229, *p* = 0.001; sadness: β = 0.118, *t(*3285) = 3.715, *p* < 0.001 (see Fig. 3B and Table S4 in the Supplemental Material).

#### Link between confidence and emotion recognition

##### Social anxiety and autistic traits

When exploring the link between confidence and accuracy in emotion recognition in relation to the clinical trait dimensions on a trial level and by emotion category, neither of the trait dimension had a substantial impact on this link (see Tables S5 and S6 in the Supplemental Material for the entire model fits).

#### Metacognitive sensitivity

**Figure 4:**
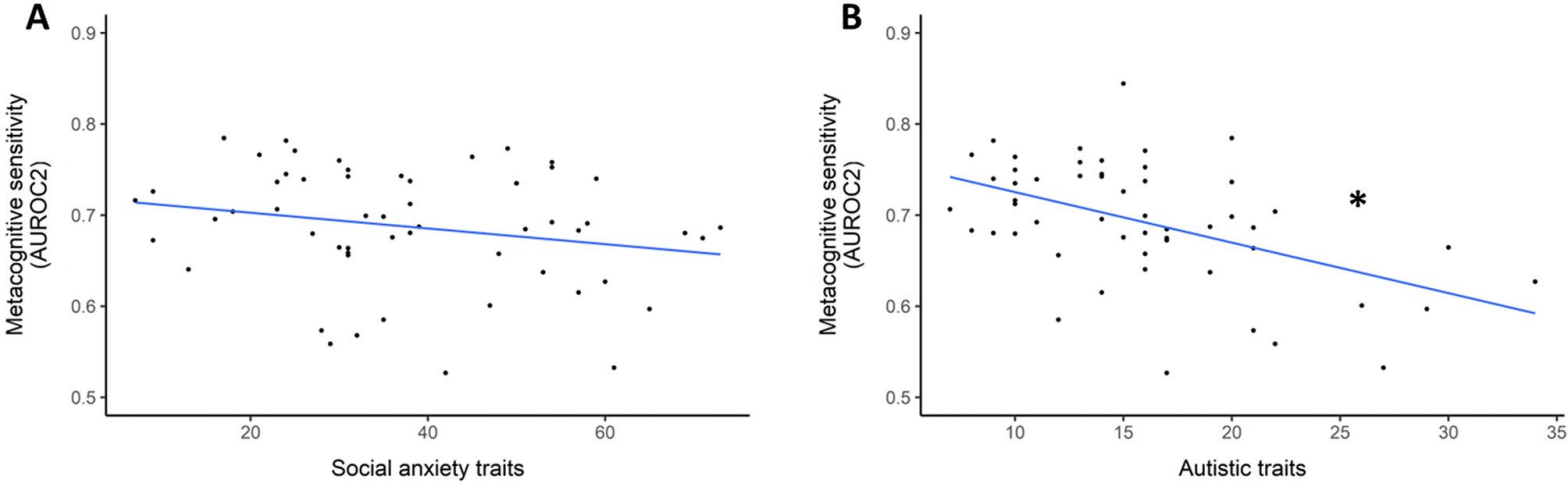
Relationship between (**A**) social anxiety traits and (**B**) autistic traits and metacognitive sensitivity, which was indexed by the area under the type 2 ROC curve (AUROC2). The blue line indicates the estimated linear relationship, with significant relationships being marked by an asterisk.

According to Mahalanobis distance measures, two participants had to be excluded from the correlation analysis between social anxiety traits and AUROC2, and three participants had to be excluded from the correlation analysis between autistic traits and AUROC2. After excluding these bivariate outliers, all distributions did not majorly deviate from normality. The two correlational analyses between the clinical trait dimension and metacognitive sensitivity (AUROC2) revealed that autistic traits, but not social anxiety traits, were significantly related to metacognitive sensitivity (see Fig. 4). As expected, metacognitive sensitivity was reduced with higher autistic traits, *r* = − 0.489, *t(*51) =  − 4.008, *p* < 0.001 and *r*_*s*_ = − 0.476, *p* < 0.001 and no significant relation was found for social anxiety, *r* = − 0.222, *t(*53) =  − 1.66, *p* = 0.103 and *r*_*s*_ = − 0.251, *p* = 0.064.

### Facial electromyography (fEMG) results

#### Facial mimicry in emotion recognition

##### Social anxiety traits

**Figure 5:**
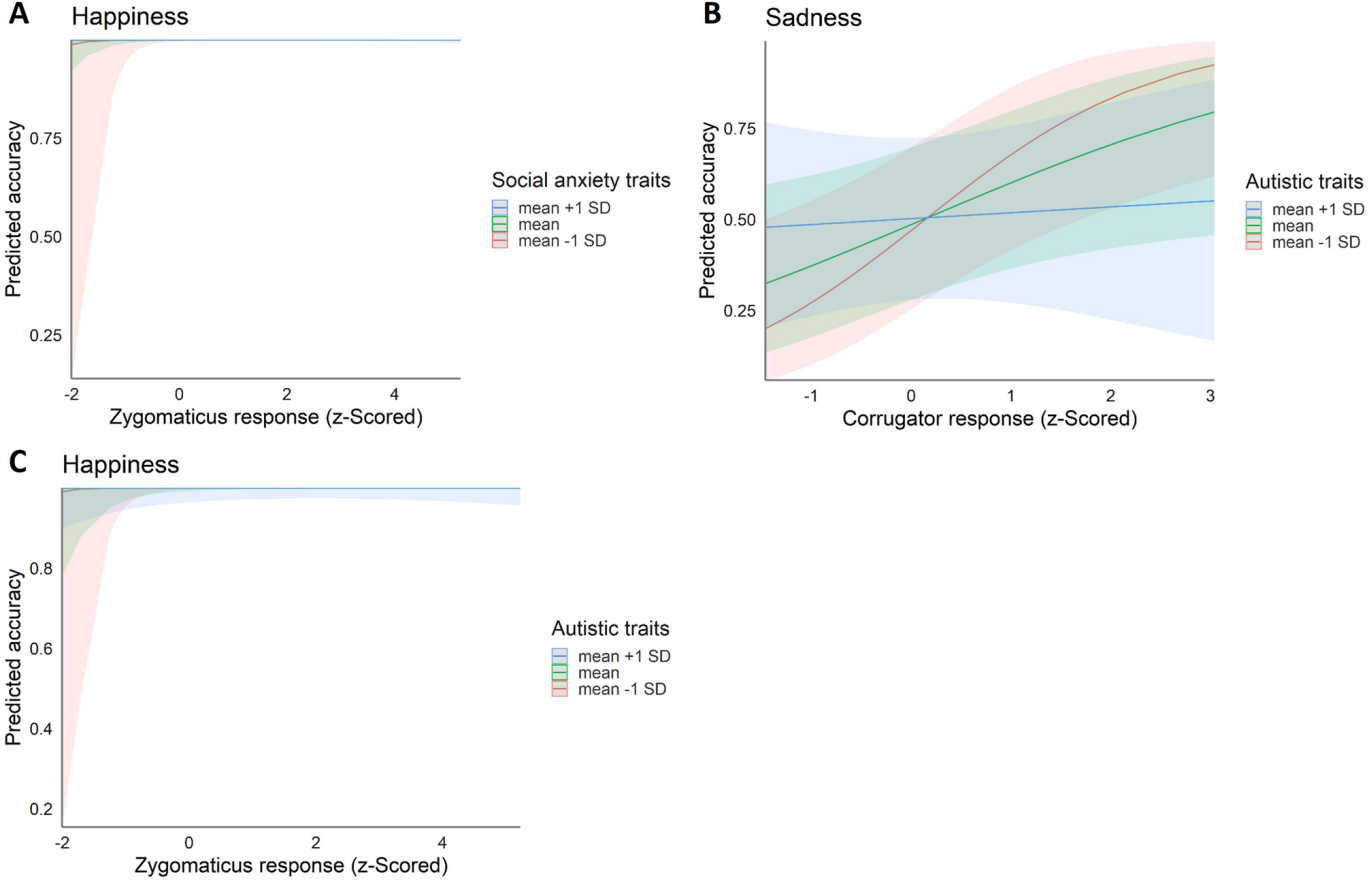
(**A**) The relationship between predicted accuracy in recognizing happy expressions and the corresponding filtered, baseline-corrected and z-scored zygomaticus activity depending on social anxiety trait levels. (**B**) The relationship between predicted accuracy in recognizing sad expressions and the corresponding filtered, baseline-corrected and z-scored Corrugator activity depending on autistic trait levels. (**C**) The relationship between predicted accuracy in recognizing happy expressions and the corresponding filtered, baseline-corrected and z-scored zygomaticus activity depending on autistic trait levels. In the model fits, accuracy was coded binomial (0–1 values). For illustrative purposes, predicted accuracies for mean values as well as mean values ± 1 SD of the continuous variables social anxiety traits and autistic traits are depicted. Shaded areas represent confidence intervals.

There was no significant interaction between corrugator activity and social anxiety traits in predicting emotion recognition accuracy of negative facial expressions, (i.e., anger, fear, and sadness, see Tables S11, S13 and S14 for the three model fits). The model on sad expressions did, however, reveal that accuracy was higher when the corrugator muscle was more strongly activated, χ^2^ (1) = 4.631, *p* = 0.031, *OR* = 1.585. Furthermore, both zygomaticus activity as well as its interaction with social anxiety traits were significant predictors in the model on happy expressions, χ^2^(1) = 4.331, *p* = 0.037, *OR* = 6.240, and χ^2^(1) = − 2.017, *p* = 0.044, *OR* = 0.213 respectively (Table S12 in the Supplemental Material). Hence, while the significant effect of zygomaticus activity hints towards a facilitating role of mimicry of smiles in emotion recognition, this link seems to be weakened with higher social anxiety traits. When examining the predicted value plot (see Fig. 5A), this effect, however, seems to be mainly driven by stronger variation in accuracies (i.e., also inaccurate responses) in individuals with lower social anxiety traits when the zygomaticus was not strongly activated. Otherwise, recognition of happy expressions was at ceiling and not much variation in relation to social anxiety trait levels could be observed.

##### Autistic traits

Similar to the models including social anxiety traits, there was no significant interaction between autistic traits and corrugator activity in accurately recognizing fearful or angry facial expressions (see Tables S15 and S17 in the Supplemental Material for the model fits). Contrasting the other negative expressions, the model on the recognition of sad expressions showed a significant interaction between corrugator activity and autistic traits as well as a significant main effect of corrugator activity. More specifically, in line with the social anxiety model, sad facial expressions were better recognized with higher corrugator activity, χ^2^(1) = 4.556, *p* = 0.033, *OR* = 1.597. This relationship was, however, weaker for higher autistic traits, interaction: χ^2^(1) = 4.142, *p* = 0.042, *OR* = 0.668 (see Fig. 5B and Table S18). Furthermore, significant effects of zygomaticus activity and of corrugator activity as well as a significant interaction between zygomaticus activity and autistic traits were found in the model on happy expressions, zygomaticus: χ^2^(1) = 5.300, *p* = 0.021, *OR* = 14.184, corrugator: χ^2^(1) = 4.679, *p* = 0.031, *OR* = 0.069, autistic traits*zygomaticus: χ^2^(1) = 5.503, *p* = 0.019, *OR* = 0.137. Thus, in addition to the activation of the zygomaticus, which was already described previously, a relaxation of the corrugator might facilitate the recognition of happy expressions. Similar to the model including social anxiety traits, autistic traits had a negative effect on the positive association between zygomaticus activity and accuracy in the recognition of happy expressions (see Fig. 5C and Table S16 in the Supplemental Material). This interaction again seemed to be driven by cases of low zygomaticus activity associated with inaccurate responses, but this time linked to low autistic trait levels.

## Discussion

In our study, we provided evidence that autistic traits and social anxiety traits are distinctly related to bottom-up (i.e., mimicry) and top-down (i.e., metacognition) components in emotion perception. Specifically, while individuals with higher social anxiety traits had significantly less confidence in their performance regarding all emotion categories, despite an unaltered actual emotion recognition performance, individuals with higher autistic traits were less accurate in the recognition of emotions, and in particular of fearful expressions. Furthermore, individuals with higher autistic traits seemed to be less able to calibrate their confidence judgments to their actual emotion recognition performance, as they displayed a poorer metacognitive sensitivity. Unexpectedly, we did not observe alterations in emotion-specific facial muscle mimicry with regard to either social anxiety or autistic traits. Yet, we found indications that mimicry of frowning, indexed by corrugator activation, might facilitate the recognition of sad expressions, whereas mimicry of smiling, indexed by zygomaticus activation (and potentially relaxation of the corrugator), might support the recognition of happy expressions. Crucially, both links were less pronounced with higher autistic traits, while there was only weak evidence for a negative effect of social anxiety traits on the link between zygomaticus activity and accuracy in recognizing happy expressions.

Contradicting our expectations, we did not find a negativity bias (i.e., an improved recognition of negative expressions) with higher social anxiety traits reflected in our main analysis on the recognition accuracy of the displayed facial expressions. When using relative accuracy scores as an outcome (see Table S20 in the Supplemental Material), however, a better recognition of angry facial expressions with higher social anxiety trait levels could be observed. Given that an improved recognition of negative expressions in SAD was also not consistently found in the literature^35^, the effect seems unstable and additional factors might play a role. For example, a heightened sensitivity to negative expressions in social anxiety (disorder) might only occur under brief exposure times or when actual interactions with the expresser could be expected^97^. In this study, the presentation time was 2 s and the participants were not engaged in any interaction. Effects related to biases in early visual attention (< 500 ms) or to the fear of being negatively judged by an interaction partner were, therefore, highly unlikely. Importantly, social anxiety traits had the expected impact on the confidence judgment with regard to emotion recognition in our study. For all expression categories, confidence was reduced with higher social anxiety traits. The underconfidence in performance did, however, not affect the general positive link between confidence in emotion recognition and actual performance. Thus, while participants seemed to be able to calibrate their confidence ratings according to their recognition performance, a relative reduction in the confidence scores might have occurred with higher social anxiety traits. This observation might be a reflection of self-related negative beliefs about one’s own social skills in high socially anxious people, which were likely formed in a public setting^66^ and translated to a more global negative social skill assessment.

Theoretical models on SAD highlight low confidence in own social performance as a relevant cognitive bias in the development and maintenance of the disorder^38, 39, 98^. Evidence for this bias has been found in various studies contrasting social performance and subjective evaluations in real-life scenarios^99–101^. The retrospective evaluation of one’s performance in a social situation, so-called post-event processing, has been especially suggested to contribute to negative beliefs about one’s social skills^102^. In both highly socially anxious individuals^103^ and individuals with a SAD diagnosis^104^, negatively-biased post-event processing has been shown to be more frequent, and positively related to social anxiety (symptoms). The lower confidence in emotion recognition associated with higher social anxiety traits in our study might also arise from doubts in one’s own ability to recognize another person’s emotional state correctly.

Facial muscle responses to emotional expressions were not found to be altered depending on social anxiety traits in our sample. This suggests that not only explicit emotion labelling but also implicit, automatic processes, namely facial mimicry, seem to be comparable across varying levels of social anxiety traits. In our study, there was also little evidence to assume that the link between facial muscle activity and emotion recognition accuracy would be modulated by social anxiety traits. The weakened positive association between zygomaticus responses and the accurate labelling of happy expressions with higher social anxiety trait levels in our study was most likely due to stronger variability in accuracies (i.e., also inaccurate responses despite close to ceiling performance overall) when zygomaticus activity was low. Additionally, the effect could not be reproduced in the analyses with relative accuracy as an outcome (see Supplemental Material S1). Taken our findings related to social anxiety traits together, heightened social anxiety trait levels were not associated with poorer emotion recognition performance or alterations in the link between facial mimicry and emotion recognition. Yet, confidence in emotion recognition was lower with higher social anxiety trait levels, which indicates that negative beliefs about one’s skills might also exist in the domain of emotion recognition. In order to overcome this and other cognitive biases, Metacognitive Training can be a useful tool in the treatment of SAD^105^.

In line with previous studies describing worse performance in emotion recognition tasks associated with ASD, we observed overall reduced accuracies with higher autistic traits, which became most apparent for fearful expressions^46–48^. The recognition of sad expressions, on the other hand, was not as strongly affected by autistic traits in the main analysis, and even improved with higher autistic trait levels in the relative accuracy analysis (see Supplemental Material S1). This observation is compatible with a previous study, which reported a better recognition of sad facial expressions with higher autistic trait levels^106^. Confidence in recognizing displays of sadness as well as fear was rated higher with higher autistic traits. Neutral and happy facial expressions, in contrast, received lower confidence ratings with higher autistic trait levels. Given the negative impact of autistic trait levels on the recognition of fear displays, higher confidence ratings seem particularly surprising. The examination of the relationship between confidence and accuracy in emotion recognition (i.e., metacognitive sensitivity) revealed, however, that participants with higher autistic trait levels in our study were less able to scale their confidence according to their actual performance. Previous research on alterations in metacognitive judgments in ASD has already described a complex pattern of both over- and underconfidence in the social-cognitive domain^69^.

Our hypothesis that higher autistic trait level would result in reduced facial mimicry responses was also not confirmed. Even though we did not explicitly instruct participants to mimic, individuals with higher autistic trait levels seemed to automatically show unaltered facial muscle activation patterns, contradicting findings in clinical populations^59^ as well as in healthy individuals with high autistic traits^62^. Importantly, it has been suggested that mimicry in ASD might especially be reduced for shorter presentation durations^107^ and occur with a delay rather than not at all^108^, which we did not examine in our study. We did, however, observe a modulation in the link between facial mimicry and emotion recognition by autistic traits. In the recognition of sad expressions, increased activity of the corrugator, indicating mimicry of sadness, was less predictive of accurate recognition whereas the same applied to stronger zygomaticus activity in the recognition of happiness. While a sole evaluation of the latter effect would be difficult due to the ceiling performance in happiness recognition (see previous paragraph) as well as to a lack of reproducibility of a result when using a relative accuracy score (see Supplemental Material S1), the robust results concerning sadness recognition support the presence of a meaningful modulation.

It seems, thus, that facial mimicry plays a less informative role in emotion recognition, at least of sad expressions, in association with higher autistic trait levels. This observation is in line with past research that did not find an effect of automatic, intentional or externally induced mimicry on reports of the participant’s own emotional experience in individuals on the autism spectrum, while neurotypical participants were considerably influenced by mimicry^109^. According to the idea of the existence of two routes in emotion recognition, a fast one involving proprioceptive (bottom-up) information and a long one involving knowledge-based (top-down) information^26^, the fast route might have been less employed in the recognition of sadness in participants with higher autistic traits. Since recognition performance of particularly sad expression was less negatively affected by higher autistic traits, judgments via the alternative, long route could have resulted in similarly successful judgments.

Previous studies have already reported qualitative differences in the recognition of sadness compared to other emotion expressions related to ASD. For example, recognition of sadness in static faces compared to point-light-displays has been found to be only improved in individuals with low but not with high autistic traits^51^. Moreover, while dynamic information (i.e., videos) generally improved emotion recognition for both autistic and neurotypical individuals, individuals on the autism spectrum recognized dynamic sad expressions worse compared to static ones^110^. Information that facilitates the recognition of sadness in neurotypical individuals might not serve individuals on the autism spectrum in the same way. Why this is specifically the case for sadness should be investigated in future studies.

Taken our findings related to autistic traits together, feedback from multiple sources might not be integrated beneficially in emotion recognition. On the one hand, confidence in emotion recognition does not seem to be scaled to actual performance. Internal feedback, in other words, the “feeling” how well one performed, might not be informative of actual performance in autism and, thus, cannot assist successful learning. Our findings suggest that, on the other hand, a simulation of observed expression might not be as informative for emotion processing in ASD compared to a neurotypical population. This claim is supported by research showing a reduced access to bodily signals (i.e., interoceptive accuracy) next to a heightened sensitivity to those signals in autism^111^, which seems to be driven by comorbid alexithymia^112, 113^. Consequently, while interventions targeting metacognitive abilities could help overcome the gap between actual performance and subjective judgments in individuals on the autism spectrum, a training focusing on the integration of information from the bodily component of an emotional experience could indirectly benefit emotion recognition and other social skills.

In addition to the results specific to the trait dimensions, our findings also add to the current discussion on the general role of facial mimicry in emotion recognition. Recent meta-analyses have described no robust relationship between facial mimicry and emotion recognition^28^, as well as broader affective judgments^29^. Our study, in contrast, revealed a link between facial mimicry responses to happy and sad expressions and associated recognition accuracy. More specifically, stronger activation of the zygomaticus and relaxation of the corrugator predicted better recognition of happiness, and stronger activation of the corrugator predicted better recognition of sadness. In some instances, sensorimotor simulation (i.e., activating facial muscle patterns that are congruent to observed emotional facial expressions) might indeed become a relevant mechanism in understanding others’ emotions. Similar to previous literature, the effects were not robust in our study (see relative accuracy analysis in Supplemental Material S1), varied depending on clinical trait levels (see paragraphs above), and we did not observe significant relationships for all expression categories (e.g., not for anger). Our study therefore corroborates evidence that an embodiment of observed emotional expressions does not seem necessary for a successful recognition^114^. Yet, facial feedback can become informative under certain conditions^29^, and our results highlight that individual differences should additionally be considered.

Despite our efforts to create a more naturalistic emotion recognition scenario by displaying spontaneous, dynamic facial expressions of emotion, participants still observed standardized stimuli in a controlled lab setting in our study. This limits the generalizability of our results as the interpretation of emotional expressions has been shown to be highly context-dependent^115, 116^. In contrast to natural scenarios, the same stimuli were also presented repeatedly (three times) in different blocks. While the repeated presentation allowed us to obtain EMG responses without priming participants with emotion category words, learning effects might have occurred. For example, emotion recognition could have been facilitated or expressions could have been perceived as less intense. More importantly, our study did not involve a real social context. Without an interaction partner who receives and responds to expressions from the participant, the social communicative function of emotional expressions, including a bidirectional coordination of affective states^3^, may get lost^117^. This limitation might also affect trait dimension-specific modulations in emotion perception. For example, in a real social situation, higher social anxiety levels have been associated with an enhanced mimicry of polite, but not enjoyment smiles^16^. Furthermore, ASD was argued to specifically become apparent in alterations in interpersonal dynamics (i.e., during bidirectional information exchange)^118, 119^. Consequently, future studies on emotion perception should be conducted in real social situations that allow for reciprocity and affect coordination.

In addition to that, even though our observations on the impact of trait levels can give us hints with regard to alterations in clinical populations, we still collected data from a non-clinical sample. Once clinical symptoms that have a severe impact on an individual’s life come into play, emotion processing might be altered differently, both qualitatively and quantitatively. Half of the participants in our sample had social anxiety trait levels that are considered clinically relevant (i.e., above 30; see Questionnaires section). While these high social anxiety trait levels for non-diagnosed individuals might result in findings that are comparable to clinical populations, this might be less applicable for our results regarding autistic traits. For example, while sadness recognition was observed to be least impacted by autistic trait levels in our study, a reduced perceptual sensitivity has been specifically described for sad facial expressions in individuals on the autism spectrum^120^. This emotion-specific recognition impairment has been shown to extend to difficulties in interpreting sadness from animations, which, in turn, has been related to worse daily social functioning in individuals on the autism spectrum^121^. Thus, in order to provide meaningful insights, results from studies including healthy participants with variations on clinical trait dimensions should always be confirmed in clinical populations as well as related to actual day-to-day experiences.

Moreover, while our sample was not gender-balanced, gender differences in mimicry and its integration in emotion recognition have been reported in past research^26, 122^, as well as in autistic traits and social anxiety traits^90, 123^. Given the predominance of female participants in our sample, our findings cannot be easily generalized to the male population. Future studies should therefore examine whether similar effects to the ones described in the current study can be observed in a more balanced or even exclusively male sample. Lastly, as we did neither manipulate facial mimicry nor metacognition, our study does not allow for causality assumptions in their role in emotion recognition. Within an emotion processing context, information is likely to flow bidirectionally and recent findings support a context-dependent influence of emotion recognition on facial mimicry^124^. Furthermore, a more fine-grained investigation of potential mediatory processes in the course of emotion perception and interpretation, such as the integration of interoceptive information^24, 125^, might benefit the understanding of variability in emotion processing and enable the formalization of testable theoretical models^126^.

In conclusion, our study provides evidence for distinct modulations of facial mimicry and metacognitive judgments in emotion recognition by autistic traits and social anxiety traits in a majorly female sample. Higher social anxiety traits were predominantly related to an underconfidence in emotion recognition, despite an unaltered performance, whereas higher autistic traits were associated with an overall worse recognition performance as well as a poorer calibration of performance judgments, and a less pronounced link between facial mimicry and emotion recognition. These trait dimension-specific patterns might also translate to the linked clinical disorders, which, however, still has to be confirmed in future studies. Importantly, particularities in processing others’ emotions have been shown to contribute to social interactions difficulties experienced by individuals on the autism spectrum and by individuals with SAD. Hence, evidence-based interventions targeting condition-specific alterations in distinct components (i.e., metacognitive beliefs and bodily feedback) hold the promise to facilitate daily social encounters and improve the quality of life in the two clinical populations.

## Supplementary Information

Supplementary Information.

## Data Availability

Following the university policy, all data and code from this project will be made available on the DataverseNL repository upon paper publication (10.34894/8UBNPL).
