# Supplementary material for: Facial mimicry and metacognitive judgments in emotion recognition are distinctly modulated by social anxiety and autistic traits
Source: Sci Rep. 2023 Jun 15;13:9730. doi: 10.1038/s41598-023-35773-6 (PMC10272184; doi:10.1038/s41598-023-35773-6)
Supplement: Supplementary file 1 — Supplementary Information. [file 41598_2023_35773_MOESM1_ESM.pdf]

Supplemental Material for

Facial mimicry and metacognitive judgments in emotion recognition are distinctly modulated by social anxiety and autistic traits

Julia Folz[1,2], Rüya Akdağ[1,2], Milica Nikolić[1,2,3], Henk van Steenbergen[1,2], Mariska E. Kret[1,2]

[1]Department of Cognitive Psychology, Institute of Psychology, Leiden University

[2]Leiden Institute for Brain and Cognition (LIBC), Leiden University

[3]Research Institute of Child Development and Education, University of Amsterdam

Corresponding author: Julia Folz

Department of Cognitive Psychology, Institute of Psychology, Leiden University

Wassenaarseweg 52, 2333 AK Leiden, The Netherlands

Phone: +31 71 527 3922

E-mail: j.folz@fsw.leidenuniv.nl

Distribution of the clinical trait score variables

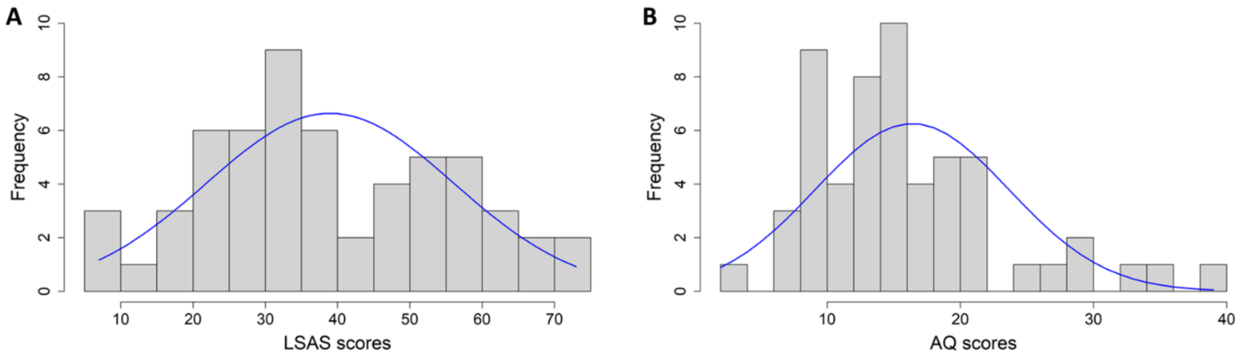

Fig. S1. Distribution of (A) social anxiety traits (LSAS scores) and (B) autistic traits (AQ scores) in our sample. The blue line indicates the estimated normal distribution, based on the mean scores, standard deviations, minimum and maximum.

Descriptive tables

Table S0. Descriptive statistics of the accuracies and relative accuracies (scaled) by emotion category (N = 57 subjects).

	Accuracy				Relative Accuracy (scaled)			
Emotion category	Mean	SD	Min	Max	Mean	SD	Min	Max
Neutral	0.89	0.14	0.5	1	0.53	0.4	-0.38	1.08
Anger	0.76	0.2	0.17	1	-0.15	0.48	-1.33	0.74
Fear	0.25	0.25	0	0.88	-0.83	0.54	-1.76	0.31
Happiness	0.97	0.08	0.57	1	0.68	0.26	-0.29	1.05
Sadness	0.51	0.23	0	1	-0.61	0.47	-1.57	0.52
Surprise	0.91	0.12	0.6	1	0.38	0.33	-0.41	0.96

Tables with model fits (main analysis)

Table S1. Results of the binomial generalized linear mixed-effects model predicting emotion recognition accuracy by emotion category, social anxiety traits and their interaction

Predictors	*Odds Ratios*	*CI*	$\chi2$	*z*	*p*
Intercept	4.382	2.788 – 6.889	41.001	6.403	**<0.001**
Emotion Category			702.880		**<0.001**

Anger	0.855	0.688 – 1.063		-1.413	0.158
Fear	0.066	0.052 – 0.083		-23.445	**<0.001**
Happiness	10.834	7.030 – 16.696		10.797	**<0.001**
Sadness	0.232	0.190 – 0.283		-14.336	**<0.001**
Surprise	3.027	2.280 – 4.020		7.654	**<0.001**
Neutral[†]	2.337	1.804 - 3.028		6.420*	**<0.001**
Social anxiety traits	1.078	0.898 – 1.294	0.655	0.809	0.418
Emotion Category*Social anxiety traits			5.456		0.363
Anger*Social anxiety traits	1.195	0.960 – 1.487		1.597	0.110
Fear*Social anxiety traits	1.078	0.866 – 1.340		0.671	0.502
Happiness*Social anxiety traits	0.931	0.609 – 1.424		-0.331	0.741
Sadness*Social anxiety traits	1.109	0.912 – 1.349		1.033	0.301
Surprise* Social anxiety traits	0.817	0.621 – 1.074		-1.449	0.147
Neutral*Social anxiety traits[†]	0.921	0.714 - 1.190		-0.553	0.580

Random Effects

σ^2	3.29
$\tau_{00\ ID}$	0.26
$\tau_{00\ StimIdentity}$	0.44
ICC	0.18
N_{ID}	57
$N_{StimIdentity}$	10
Observations	3176
Marginal R^2 / Conditional R^2	0.418 / 0.521

[†]taken from general linear hypothesis calculation

Table S2. Results of the binomial generalized linear mixed-effects model predicting emotion recognition accuracy by emotion category, autistic traits and their interaction

Predictors	*Odds Ratios*	*CI*	$\chi2$	*z*	*p*
Intercept	4.438	2.811 – 7.007	40.888	6.394	**<0.001**
Emotion Category			666.374		**<0.001**
Anger	0.828	0.664 – 1.034		-1.667	0.096
Fear	0.059	0.046 – 0.075		-22.639	**<0.001**
Happiness	11.894	7.391 – 19.142		10.200	**<0.001**
Sadness	0.232	0.189 – 0.285		-13.922	**<0.001**
Surprise	3.045	2.279 – 4.069		7.528	**<0.001**
Neutral†	2.433	1.857 - 3.187		6.459	**<0.001**
Autistic traits	0.763	0.639 – 0.910	8.985	-2.998	**0.003**
Emotion Category*Autistic traits			21.606		**0.001**
Anger*Autistic traits	1.106	0.895 – 1.367		0.936	0.349
Fear*Autistic traits	0.637	0.488 – 0.833		-3.292	**0.001**
Happiness*Autistic traits	0.782	0.543 – 1.127		-1.319	0.187
Sadness*Autistic traits	1.322	1.089 – 1.605		2.821	**0.005**
Surprise*Autistic traits	1.341	1.015 – 1.771		2.067	**0.039**
Neutral*Autistic traits†	1.022	0.796 - 1.314		0.176	0.860

**Random Effects**	
σ^2	3.29
$\tau_{00\ ID}$	0.25
$\tau_{00\ StimIdentity}$	0.45
ICC	0.17
N_{ID}	56
$N_{StimIdentity}$	10
Observations	3124
Marginal R^2 / Conditional R^2	0.445 / 0.542

†taken from general linear hypothesis calculation

Table S3. Results of the linear mixed-effects model predicting confidence in emotion recognition by emotion category, social anxiety traits and their interaction

Predictors	β	*CI*	*F*	*t*	*p*
Intercept	0.000	-0.182 – 0.182	0.000	0.000	1.000
Emotion Category			118.666		**<0.001**
Anger	-0.247	-0.308 – -0.186		-7.932	**<0.001**
Fear	-0.287	-0.348 – -0.226		-9.209	**<0.001**
Happiness	0.614	0.552 – 0.675		19.695	**<0.001**
Sadness	-0.356	-0.417 – -0.295		-11.438	**<0.001**
Surprise	0.084	0.023 – 0.145		2.699	**0.007**
Neutral†	0.193	0.132 - 0.254		6.186	**<0.001**
Social anxiety traits	-0.132	-0.246 – -0.018	5.362	-2.316	**0.024**
Emotion Category*Social anxiety traits			0.796		0.552
Anger*Social anxiety traits	0.043	-0.018 – 0.105		1.379	0.168
Fear*Social anxiety traits	-0.043	-0.104 – 0.019		-1.355	0.175
Happiness*Social anxiety traits	0.027	-0.035 – 0.088		0.844	0.399
Sadness*Social anxiety traits	-0.006	-0.068 – 0.056		-0.194	0.846
Surprise* Social anxiety traits	-0.005	-0.067 – 0.057		-0.162	0.871
Neutral* Social anxiety traits†	-0.016	-0.078 - 0.046		-0.512	0.609

Random Effects

σ^2	0.66
$\tau_{00\ ID}$	0.17
$\tau_{00\ StimIdentity}$	0.05
ICC	0.25
$N_{StimIdentity}$	10
N_{ID}	57
Observations	3420
Marginal R^2 / Conditional R^2	0.131 / 0.344

†taken from general linear hypothesis calculation, z-score instead of t-value

Table S4. Results of the linear mixed-effects model predicting confidence in emotion recognition by emotion category and autistic traits

Predictors	β	*CI*	*F*	*t*	*p*
Intercept	-0.008	-0.194 – 0.179	0.007	-0.086	0.932
Emotion Category			118.164		**<0.001**
Anger	-0.251	-0.312 – -0.189		-7.993	**<0.001**
Fear	-0.289	-0.351 – -0.227		-9.206	**<0.001**
Happiness	0.615	0.554 – 0.677		19.605	**<0.001**
Sadness	-0.357	-0.418 – -0.295		-11.361	**<0.001**
Surprise	0.083	0.021 – 0.144		2.630	**0.009**
Neutral†	0.199	0.137 - 0.260		6.325	**<0.001**
Autistic traits	-0.019	-0.140 – 0.101	0.103	-0.320	0.750
Emotion Category*Autistic traits			9.531		**<0.001**
Anger*Autistic traits	0.012	-0.050 – 0.074		0.378	0.705
Fear*Autistic traits	0.102	0.040 – 0.164		3.229	**0.001**
Happiness*Autistic traits	-0.104	-0.166 – -0.042		-3.271	**0.001**
Sadness*Autistic traits	0.118	0.056 – 0.180		3.715	**<0.001**
Surprise*Autistic traits	0.019	-0.043 – 0.081		0.611	0.541
Neutral*Autistic traits†	-0.148	-0.210 - -0.088		-4.663	**<0.001**

Random Effects

σ^2	0.66
$\tau_{00\ ID}$	0.19
$\tau_{00\ StimIdentity}$	0.05
ICC	0.26
$N_{StimIdentity}$	10
N_{ID}	56
Observations	3360
Marginal R^2 / Conditional R^2	0.124 / 0.352

†taken from general linear hypothesis calculation, z-score instead of t-value

Table S5. Results of the binomial generalized linear mixed-effects model predicting emotion recognition accuracy by emotion category, social anxiety traits, confidence and all possible interactions

Predictors	*Odds Ratios*	*CI*	$\chi 2$	*z*	*p*
Intercept	4.779	3.145 – 7.262	53.715	7.329	**<0.001**
Confidence	1.922	1.668 – 2.215	81.745	9.041	**<0.001**
Emotion Category			605.949		**<0.001**
Anger	1.239	0.935 – 1.642		1.494	0.135
Fear	0.065	0.051 – 0.083		-22.207	**<0.001**
Happiness	8.808	5.351 – 14.499		8.556	**<0.001**
Sadness	0.256	0.206 – 0.319		-12.213	**<0.001**
Surprise	2.636	1.971 – 3.526		6.531	**<0.001**
Neutral†	2.085	1.582 - 2.751		5.206	**<0.001**
Social anxiety traits	1.172	0.964 – 1.425	2.544	1.595	0.111
Emotion Category*Confidence			23.616		**<0.001**
Anger*Confidence	1.432	1.113 – 1.842		2.796	**0.005**
Fear*Confidence	0.688	0.544 – 0.871		-3.117	**0.002**
Happiness*Confidence	1.590	1.025 – 2.465		2.072	**0.038**
Sadness*Confidence	0.976	0.786 – 1.213		-0.217	0.829
Surprise*Confidence	0.690	0.499 – 0.955		-2.241	**0.025**
Neutral*Confidence†	0.946	0.726 - 1.234		-0.405	0.686
Confidence*Social anxiety traits	0.998	0.862 – 1.156	0.000	-0.021	0.983
Emotion Category*Social anxiety traits			4.781		0.443
Anger*Social anxiety traits	1.215	0.920 – 1.606		1.370	0.171
Fear*Social anxiety traits	1.036	0.822 – 1.306		0.299	0.765
Happiness*Social anxiety traits	0.995	0.614 – 1.611		-0.022	0.982
Sadness*Social anxiety traits	1.077	0.870 – 1.334		0.682	0.495
Surprise* Social anxiety traits	0.787	0.595 – 1.042		-1.670	0.095
Neutral*Social anxiety traits†	0.942	0.717 - 1.236		-0.433	0.665

Emotion Category*Confidence*Social anxiety traits			2.442		0.785
Anger*Confidence*Social anxiety traits	0.903	0.701 – 1.163		-0.788	0.430
Fear*Confidence*Social anxiety traits	0.979	0.774 – 1.239		-0.174	0.862
Happiness*Confidence*Social anxiety traits	1.324	0.821 – 2.133		1.152	0.249
Sadness*Confidence*Social anxiety traits	0.903	0.727 – 1.121		-0.924	0.355
Surprise*Confidence*Social anxiety traits	0.895	0.656 – 1.220		-0.703	0.482
Neutral*Confidence*Social anxiety traits†	1.058	0.809 - 1.381		0.407	0.684

Random Effects

σ^2	3.29
$\tau_{00\ ID}$	0.28
$\tau_{00\ StimIdentity}$	0.35
ICC	0.16
N_{ID}	57
$N_{StimIdentity}$	10
Observations	3176
Marginal R^2 / Conditional R^2	0.495 / 0.576

†taken from general linear hypothesis calculation, z-score instead of t-value

Table S6. Results of the binomial generalized linear mixed-effects model predicting emotion recognition accuracy by emotion category, autistic traits, confidence and all possible interactions

Predictors	*Odds Ratios*	*CI*	χ2	*z*	*p*
Intercept	4.827	3.151 – 7.395	52.325	7.234	**<0.001**
Confidence	1.910	1.654 – 2.206	77.682	8.814	**<0.001**
Emotion Category			582.327		**<0.001**
Anger	1.212	0.916 – 1.603		1.344	0.179

Fear	0.058	0.045 – 0.075		-21.496	**<0.001**
Happiness	9.361	5.477 – 15.998		8.179	**<0.001**
Sadness	0.260	0.208 – 0.325		-11.842	**<0.001**
Surprise	2.729	2.027 – 3.676		6.611	**<0.001**
Neutral†	2.140	1.608 - 2.846		5.218	**<0.001**
Autistic traits	0.743	0.613 – 0.901	9.097	-3.016	**0.003**
Emotion Category*Confidence			23.014		**<0.001**
Anger*Confidence	1.377	1.074 – 1.765		2.522	**0.012**
Fear*Confidence	0.657	0.514 – 0.841		-3.340	**0.001**
Happiness*Confidence	1.628	1.042 – 2.546		2.139	**0.032**
Sadness*Confidence	0.968	0.779 – 1.202		-0.296	0.767
Surprise*Confidence	0.712	0.516 – 0.982		-2.073	**0.038**
Neutral*Confidence†	0.985	0.755 - 1.287		-0.108	0.914
Confidence * Autistic traits	0.883	0.778 – 1.002	3.697	-1.923	0.055
Emotion Category*Autistic traits			21.470		**0.001**
Anger*Autistic traits	1.064	0.818 – 1.383		0.460	0.646
Fear*Autistic traits	0.575	0.424 – 0.780		-3.553	**<0.001**
Happiness*Autistic traits	0.819	0.556 – 1.205		-1.013	0.311
Sadness*Autistic traits	1.353	1.090 – 1.679		2.742	**0.006**
Surprise*Autistic traits	1.338	1.001 – 1.788		1.967	**0.049**
Neutral*Autistic traits†	1.103	0.850 - 1.432		0.737	0.461
Emotion Category*Confidence*Autistic traits			7.763		0.170
Anger*Confidence*Autistic traits	1.130	0.889 – 1.436		0.998	0.318
Fear*Confidence*Autistic traits	0.865	0.655 – 1.143		-1.019	0.308
Happiness*Confidence*Autistic traits	0.874	0.638 – 1.197		-0.839	0.402
Sadness*Confidence*Autistic traits	1.276	1.037 – 1.570		2.299	**0.022**
Surprise*Confidence*Autistic traits	1.033	0.767 – 1.392		0.216	0.829
Neutral*Confidence*Autistic traits†	0.888	0.693 - 1.138		-0.942	0.346

Random Effects

σ^2	3.29
$\tau_{00\ ID}$	0.29
$\tau_{00\ StimIdentity}$	0.36
ICC	0.16
N $_{ID}$	56
N $_{StimIdentity}$	10
Observations	3124
Marginal R^2 / Conditional R^2	0.521 / 0.600

†taken from general linear hypothesis calculation, z-score instead of t-value

Table S7. Results of the linear model predicting the category-averaged Corrugator activity by emotion category, social anxiety traits and their interaction.

Predictors	β	*CI*	*F*	*t*	*p*
Intercept	0.094	0.035 – 0.154	9.708	3.116	**0.002**
Emotion Category			76.338		**<0.001**
Anger	0.117	0.033 – 0.201		2.740	**0.007**
Fear	-0.037	-0.121 – 0.047		-0.863	0.389
Happiness	-0.547	-0.631 – -0.463		-12.806	**<0.001**
Sadness	0.036	-0.048 – 0.121		0.852	0.395
Social anxiety traits	0.001	-0.059 – 0.061	0.002	0.043	0.966
Emotion Category*Social anxiety traits			0.387		0.818
Anger*Social anxiety traits	0.016	-0.069 – 0.101		0.367	0.714
Fear*Social anxiety traits	0.000	-0.084 – 0.085		0.011	0.991
Happiness*Social anxiety traits	-0.034	-0.119 – 0.051		-0.796	0.427
Sadness*Social anxiety traits	-0.015	-0.100 – 0.070		-0.355	0.723
Observations	285				
R^2 / R^2 adjusted	0.528 / 0.512				

†Note: The neutral category was taken as reference level in this analysis.

Table S8. Results of the linear model predicting the category-averaged Corrugator activity by emotion category, autistic traits and their interaction.

Predictors	β	*CI*	*F*	*t*	*p*
Intercept	0.093	0.033 – 0.153	9.455	3.075	**0.002**
Emotion Category			77.810		**<0.001**
Anger	0.122	0.037 – 0.206		2.844	**0.005**
Fear	-0.039	-0.123 – 0.045		-0.918	0.359
Happiness	-0.551	-0.635 – -0.466		-12.876	**<0.001**
Sadness	0.041	-0.044 – 0.125		0.950	0.343
Autistic traits	-0.014	-0.074 – 0.046	0.201	-0.448	0.654
Emotion Category*Autistic traits			1.842		0.121
Anger*Autistic traits	-0.000	-0.085 – 0.085		-0.005	0.996
Fear*Autistic traits	0.028	-0.057 – 0.113		0.655	0.513
Happiness*Autistic traits	0.095	0.010 – 0.180		2.192	**0.029**
Sadness*Autistic traits	-0.002	-0.087 – 0.083		-0.055	0.956
Observations	342				
R^2 / R^2 adjusted	0.526 / 0.511				

†Note: The neutral category was taken as reference level in this analysis.

Table S9. Results of the bootstrapping analysis associated with the linear model predicting the category-averaged zygomaticus activity by emotion category (happiness vs. neutral), social anxiety traits and their interaction.

Predictors	*B*	*CI*	*p*
Intercept	-0.077	-0.118 – -0.034	**0.002**
Emotion Category: Happiness	0.540	0.391 – 0.705	**<0.001**
Social anxiety traits	-0.019	-0.067 – 0.026	0.374
Emotion Category: Happiness*Social anxiety traits	0.126	-0.040 – 0.288	0.116
Observations	114		
R^2 / R^2 adjusted	0.296 / 0.277		

†Note: The neutral category was taken as reference level in this analysis.

Table S10. Results of the bootstrapping analysis associated with the linear model predicting the category-averaged zygomaticus activity by emotion category (happiness vs. neutral), autistic traits and their interaction.

Predictors	*B*	*CI*	*p*
Intercept	-0.079	-0.121 – -0.032	**0.002**
Emotion Category: Happiness	0.552	0.402 – 0.731	**<0.001**
Autistic traits	0.002	-0.038 – 0.049	0.902
Emotion Category: Happiness *Autistic traits	-0.052	-0.180 – 0.086	0.436
Observations	112		
R^2 / R^2 adjusted	0.290 / 0.271		

†Note: The neutral category was taken as reference level in this analysis.

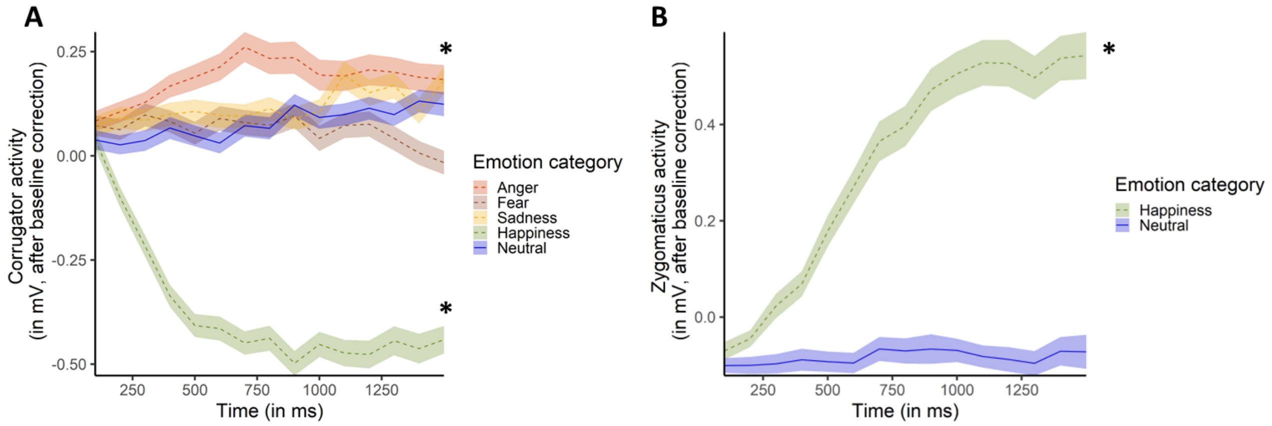

Fig. S2. Time course of the filtered, baseline-corrected and z-scored facial electromyography (EMG) signal over (A) the Corrugator Supercilii region and (B) the Zygomaticus Major region related to the passive viewing of the naturalistic facial expressions by emotion category. The mean of the first 500ms of each 2s video (neutral expression) was taken as a baseline and only the mean EMG response in the last second of stimulus presentation (full-blown expression, 500ms – 1500ms in plot) was considered in the facial EMG analysis. The coloured shaded areas around the values indicate standard errors of the means and asterisks mark statistical significance.

Table S11. Results of the binomial generalized linear mixed-effects model predicting recognition accuracy of angry facial expressions by social anxiety traits, corrugator muscle activity and the 2-way interaction between social anxiety traits and corrugator muscle activity

Predictors	*Odds Ratios*	*CI*	χ^2	*z*	*p*
Intercept	6.329	2.251 – 17.795	12.236	3.498	**<0.001**
Social anxiety traits	1.409	0.911 – 2.178	2.373	1.540	0.123
Corrugator activity	0.722	0.455 – 1.146	1.192	-1.383	0.167
Corrugator activity* Social anxiety traits	1.399	0.857 – 2.283	1.806	1.344	0.179
**Random Effects**					
σ^2	3.29				
$\tau_{00\ ID}$	1.58				
$\tau_{00\ StimIdentity}$	2.22				
ICC	0.54				
N_{ID}	57				
$N_{StimIdentity}$	10				
Observations	523				
Marginal R^2 / Conditional R^2	0.033 / 0.551				

Table S12. Results of the binomial generalized linear mixed-effects model predicting recognition accuracy of happy facial expressions by social anxiety traits, zygomaticus muscle activity, corrugator muscle activity and the 2-way interactions between social anxiety traits and each muscles activity

Predictors	*Odds Ratios*	*CI*	χ^2	*z*	*p*
Intercept	38866.002	371.382 – 4067421.073	19.836	4.454	**<0.001**
Zygomaticus activity	6.240	1.112 – 34.998	4.331	2.081	**0.037**
Social anxiety traits	0.998	0.175 – 5.693	0.000	-0.002	0.998
Corrugator activity	0.379	0.076 – 1.900	1.391	-1.179	0.238
Zygomaticus activity* Social anxiety traits	0.213	0.047 – 0.957	4.068	-2.017	**0.044**
Corrugator activity* Social anxiety traits	1.339	0.266 – 6.734	0.126	0.355	0.723
**Random Effects**					
σ^2	3.29				
$\tau_{00\ ID}$	47.76				
$\tau_{00\ StimIdentity}$	3.88				

ICC	0.94
N $_{ID}$	57
N $_{StimIdentity}$	10
Observations	556
Marginal R^2 / Conditional R^2	0.087 / 0.945

Table S13. Results of the binomial generalized linear mixed-effects model predicting recognition accuracy of fearful facial expressions by social anxiety traits, corrugator muscle activity and the 2-way interaction between social anxiety traits and corrugator muscle activity

Predictors	*Odds Ratios*	*CI*	χ^2	*z*	*p*
Intercept	0.224	0.127 – 0.395	26.687	-5.166	**<0.001**
Social anxiety traits	1.170	0.750 – 1.825	0.480	0.693	0.488
Corrugator activity	0.676	0.405 – 1.128	2.251	-1.500	0.134
Corrugator activity* Social anxiety traits	1.005	0.595 – 1.698	0.000	0.018	0.986
**Random Effects**					
σ^2	3.29				
$\tau_{00\ ID}$	1.90				
$\tau_{00\ StimIdentity}$	0.30				
ICC	0.40				
N $_{ID}$	57				
N $_{StimIdentity}$	10				
Observations	504				
Marginal R^2 / Conditional R^2	0.012 / 0.408				

Table S14. Results of the binomial generalized linear mixed-effects model predicting recognition accuracy of sad facial expressions by social anxiety traits, corrugator muscle activity and the 2-way interaction between social anxiety traits and corrugator muscle activity

Predictors	*Odds Ratios*	*CI*	χ^2	*z*	*p*
Intercept	0.926	0.383 – 2.236	0.030	-0.172	0.863
Social anxiety traits	1.303	0.931 – 1.823	2.385	1.544	0.122
Corrugator activity	1.585	1.042 – 2.410	4.631	2.152	**0.031**
Corrugator activity* Social anxiety traits	0.874	0.580 – 1.316	0.417	-0.646	0.519

Random Effects

σ^2	3.29
$\tau_{00\ ID}$	0.97
$\tau_{00\ StimIdentity}$	1.73
ICC	0.45
N_{ID}	57
$N_{StimIdentity}$	10
Observations	522
Marginal R^2 / Conditional R^2	0.019 / 0.459

Table S15. Results of the binomial generalized linear mixed-effects model predicting recognition accuracy of angry facial expressions by autistic traits, corrugator muscle activity and the 2-way interaction between autistic traits and corrugator muscle activity

Predictors	*Odds Ratios*	*CI*	χ^2	*z*	*p*
Intercept	6.151	2.167 – 17.462	11.645	3.412	**0.001**
Autistic traits	0.819	0.526 – 1.276	0.780	-0.883	0.377
Corrugator activity	0.748	0.485 – 1.154	1.725	-1.313	0.189
Corrugator activity* Autistic traits	0.673	0.379 – 1.196	1.822	-1.350	0.177
**Random Effects**					
σ^2	3.29				
$\tau_{00\ ID}$	1.67				
$\tau_{00\ StimIdentity}$	2.25				
ICC	0.54				
N_{ID}	56				
$N_{StimIdentity}$	10				
Observations	516				
Marginal R^2 / Conditional R^2	0.018 / 0.552				

Table S16. Results of the binomial generalized linear mixed-effects model predicting recognition accuracy of happy facial expressions by autistic traits, zygomaticus muscle activity, corrugator muscle activity and the 2-way interactions between autistic traits and each muscles activity

Predictors	*Odds Ratios*	*CI*	χ^2	*z*	*p*
Intercept	66148.235	75.866 – 57675479.197	10.324	3.213	**0.001**
Zygomaticus activity	14.184	1.483 – 135.647	5.300	2.302	**0.021**

Autistic traits	0.423	0.045 – 3.978	0.565	-0.752	0.452
Corrugator activity	0.069	0.006 – 0.778	4.679	-2.163	**0.031**
Zygomaticus activity* Autistic traits	0.137	0.026 – 0.721	5.503	-2.346	**0.019**
Corrugator activity* Autistic traits	4.806	0.930 – 24.829	3.510	1.874	0.061
**Random Effects**					
σ^2	3.29				
$\tau_{00\ ID}$	45.84				
$\tau_{00\ StimIdentity}$	5.44				
ICC	0.94				
N_{ID}	56				
$N_{StimIdentity}$	10				
Observations	546				
Marginal R^2 / Conditional R^2	0.282 / 0.957				

Table S17. Results of the binomial generalized linear mixed-effects model predicting recognition accuracy of fearful facial expressions by autistic traits, corrugator muscle activity and the 2-way interaction between autistic traits and corrugator muscle activity

Predictors	*Odds Ratios*	*CI*	χ^2	*z*	*p*
Intercept	0.212	0.122 – 0.368	30.344	-5.509	**<0.001**
Autistic traits	0.427	0.257 – 0.707	10.942	-3.308	**0.001**
Corrugator activity	0.668	0.368 – 1.213	1.756	-1.325	0.185
Corrugator activity* Autistic traits	0.882	0.406 – 1.916	0.101	-0.318	0.751
**Random Effects**					
σ^2	3.29				
$\tau_{00\ ID}$	1.51				
$\tau_{00\ StimIdentity}$	0.26				
ICC	0.35				
N_{ID}	56				
$N_{StimIdentity}$	10				
Observations	496				
Marginal R^2 / Conditional R^2	0.135 / 0.438				

Table S18. Results of the binomial generalized linear mixed-effects model predicting recognition accuracy of sad facial expressions by autistic traits, corrugator muscle activity and the 2-way interaction between autistic traits and corrugator muscle activity

Predictors	*Odds Ratios*	*CI*	χ^2	*z*	*p*
Intercept	0.944	0.387 – 2.303	0.016	-0.126	0.900
Autistic traits	1.068	0.754 – 1.513	0.138	0.371	0.710
Corrugator activity	1.597	1.039 – 2.454	4.556	2.135	**0.033**
Corrugator activity* Autistic traits	0.668	0.453 – 0.985	4.142	-2.035	**0.042**
**Random Effects**					
σ^2	3.29				
$\tau_{00\ ID}$	1.04				
$\tau_{00\ StimIdentity}$	1.75				
ICC	0.46				
N_{ID}	56				
$N_{StimIdentity}$	10				
Observations	514				
Marginal R^2 / Conditional R^2	0.017 / 0.468				

Model fits with relative emotion recognition accuracy (additional analysis)

Table S20. Results of the linear mixed-effects model predicting relative emotion recognition accuracy by emotion category, social anxiety traits and their interaction

Predictors	β	*CI*	*F*	*t*	*p*
Intercept	0.000	-0.170 – 0.170	0.000	0.000	1.000
Emotion Category			395.327		**<0.001**
Anger	-0.148	-0.204 – -0.091		-5.139	**<0.001**
Fear	-0.829	-0.886 – -0.773		-28.816	**<0.001**
Happiness	0.676	0.620 – 0.733		23.504	**<0.001**
Sadness	-0.610	-0.666 – -0.553		-21.182	**<0.001**
Surprise	0.381	0.324 – 0.437		13.235	**<0.001**
Neutral[†]	0.530	0.473 – 0.586		18.400	**<0.001**
Social anxiety traits	-0.009	-0.073 – 0.054	0.082	-0.286	0.776
Emotion Category*Social anxiety traits			2.723		**0.018**
Anger*Social anxiety traits	0.081	0.024 – 0.138		2.791	**0.005**
Fear*Social anxiety traits	0.008	-0.049 – 0.065		0.275	0.783
Happiness*Social anxiety traits	-0.028	-0.085 – 0.029		-0.964	0.335
Sadness*Social anxiety traits	0.038	-0.019 – 0.095		1.306	0.192
Surprise* Social anxiety traits	-0.053	-0.110 – 0.004		-1.834	0.067
Neutral*Social anxiety traits[†]	-0.046	-0.103 – 0.011		-1.574	0.116
**Random Effects**					
σ^2	0.57				
$\tau_{00\ ID}$	0.05				
$\tau_{00\ StimIdentity}$	0.06				
ICC	0.17				
N_{ID}	57				
$N_{StimIdentity}$	10				
Observations	3420				
Marginal R^2 / Conditional R^2	0.327 / 0.439				

[†]taken from general linear hypothesis calculation, z-score instead of t-value

Table S21. Results of the linear mixed-effects model predicting relative emotion recognition accuracy by emotion category, autistic traits and their interaction

Predictors	β	*CI*	*F*	*t*	*p*
Intercept	0.006	-0.162 – 0.174	0.006	0.068	0.946
Emotion Category			396.013		**<0.001**
Anger	-0.151	-0.208 – -0.094		-5.193	**<0.001**
Fear	-0.834	-0.890 – -0.777		-28.725	**<0.001**
Happiness	0.672	0.615 – 0.729		23.167	**<0.001**
Sadness	-0.607	-0.664 – -0.550		-20.906	**<0.001**
Surprise	0.383	0.326 – 0.440		13.201	**<0.001**
Neutral†	0.536	0.479 – 0.593		18.460	**<0.001**
Autistic traits	-0.068	-0.129 – -0.007	4.812	-2.193	**0.033**
Emotion Category*Autistic traits			4.416		**0.001**
Anger*Autistic traits	-0.008	-0.065 – 0.050		-0.266	0.791
Fear*Autistic traits	-0.104	-0.161 – -0.046		-3.547	**<0.001**
Happiness*Autistic traits	-0.015	-0.073 – 0.042		-0.523	0.601
Sadness*Autistic traits	0.097	0.040 – 0.155		3.327	**0.001**
Surprise*Autistic traits	0.044	-0.013 – 0.101		1.502	0.133
Neutral*Autistic traits†	-0.014	-0.072 – 0.043		-0.494	0.621
**Random Effects**					
σ^2	0.57				
$\tau_{00\ ID}$	0.04				
$\tau_{00\ StimIdentity}$	0.06				
ICC	0.16				
N_{ID}	56				
$N_{StimIdentity}$	10				
Observations	3360				
Marginal R^2 / Conditional R^2	0.333 / 0.440				

†taken from general linear hypothesis calculation, z-score instead of t-value

Table S22. Results of the linear mixed-effects model predicting relative emotion recognition accuracy by emotion category, social anxiety traits, confidence and all possible interactions

Predictors	β	*CI*	*F*	*t*	*p*
Intercept	0.001	-0.130 – 0.133	0.000	0.021	0.984
Confidence	0.319	0.290 – 0.348	468.387	21.688	**<0.001**
Emotion Category			278.059		**<0.001**
Anger	-0.040	-0.094 – 0.015		-1.426	0.154
Fear	-0.784	-0.840 – -0.728		-27.501	**<0.001**
Happiness	0.482	0.416 – 0.549		14.262	**<0.001**
Sadness	-0.476	-0.533 – -0.420		-16.527	**<0.001**
Surprise	0.352	0.298 – 0.406		12.795	**<0.001**
Neutral†	0.466	0.411 – 0.521		16.730	**<0.001**
Social anxiety traits	0.026	-0.032 – 0.083	0.762	0.873	0.386
Emotion Category*Confidence			12.112		**<0.001**
Anger*Confidence	0.149	0.096 – 0.201		5.572	**<0.001**
Fear*Confidence	-0.171	-0.228 – -0.114		-5.863	**<0.001**
Happiness*Confidence	0.003	-0.063 – 0.069		0.082	0.934
Sadness*Confidence	0.050	-0.006 – 0.105		1.752	0.080
Surprise*Confidence	-0.045	-0.105 – 0.016		-1.452	0.146
Neutral*Confidence†	0.014	-0.042 – 0.070		0.491	0.624
Confidence*Social anxiety traits	0.000	-0.029 – 0.029	0.001	0.031	0.974
Emotion Category*Social anxiety traits			2.968		**0.011**
Anger*Social anxiety traits	0.071	0.016 – 0.126		2.513	**0.012**
Fear*Social anxiety traits	0.006	-0.051 – 0.062		0.200	0.842
Happiness*Social anxiety traits	-0.056	-0.125 – 0.012		-1.609	0.108
Sadness*Social anxiety traits	0.062	0.005 – 0.119		2.135	**0.033**
Surprise* Social anxiety traits	-0.047	-0.102 – 0.007		-1.702	0.089
Neutral*Social anxiety traits†	-0.035	-0.091 – 0.021		-1.228	0.219

Emotion Category*Confidence*Social anxiety traits			1.869		0.096
Anger*Confidence*Social anxiety traits	-0.070	-0.123 – -0.018		-2.626	**0.009**
Fear*Confidence*Social anxiety traits	0.023	-0.033 – 0.078		0.801	0.423
Happiness*Confidence*Social anxiety traits	0.043	-0.027 – 0.112		1.205	0.228
Sadness*Confidence*Social anxiety traits	0.023	-0.033 – 0.079		0.814	0.416
Surprise*Confidence*Social anxiety traits	-0.032	-0.091 – 0.027		-1.051	0.294
Neutral*Confidence*Social anxiety traits†	0.013	-0.044 – 0.071		0.460	0.646
**Random Effects**					
σ^2	0.49				
$\tau_{00\ ID}$	0.04				
$\tau_{00\ StimIdentity}$	0.04				
ICC	0.13				
N_{ID}	57				
$N_{StimIdentity}$	10				
Observations	3420				
Marginal R^2 / Conditional R^2	0.435 / 0.511				

†taken from general linear hypothesis calculation, z-score instead of t-value

Table S23. Results of the linear mixed-effects model predicting relative emotion recognition accuracy by emotion category, autistic traits, confidence and all possible interactions

Predictors	β	*CI*	*F*	*t*	*p*
Intercept	0.004	-0.135 – 0.133	0.003	0.058	0.955
Confidence	0.322	0.293 – 0.351	472.878	21.800	**<0.001**
Emotion Category			274.689		**<0.001**
Anger	-0.031	-0.086 – 0.024		-1.119	0.263
Fear	-0.784	-0.840 – -0.728		-27.476	**<0.001**

Happiness	0.468	0.402 – 0.535		13.782	**<0.001**
Sadness	-0.476	-0.533 – -0.419		-16.445	**<0.001**
Surprise	0.364	0.310 – 0.418		13.260	**<0.001**
Neutral†	0.460	0.404 – 0.515		16.340	**<0.001**
Autistic traits	-0.057	-0.118 – -0.007	4.266	-2.065	**0.043**
Emotion Category*Confidence			10.548		**<0.001**
Anger*Confidence	0.132	0.080 – 0.185		4.928	**<0.001**
Fear*Confidence	-0.167	-0.224 – -0.110		-5.779	**<0.001**
Happiness*Confidence	0.011	-0.056 – 0.077		0.316	0.752
Sadness*Confidence	0.040	-0.015 – 0.096		1.414	0.157
Surprise*Confidence	-0.036	-0.096 – 0.023		-1.196	0.232
Neutral*Confidence†	0.020	-0.036 – 0.076		0.712	0.476
Confidence * Autistic traits	-0.024	-0.051 – 0.004	2.902	-1.707	0.088
Emotion Category*Autistic traits			5.520		**<0.001**
Anger*Autistic traits	-0.037	-0.095 – 0.021		-1.243	0.214
Fear*Autistic traits	-0.133	-0.189 – -0.077		-4.670	**<0.001**
Happiness*Autistic traits	0.026	-0.032 – 0.085		0.893	0.372
Sadness*Autistic traits	0.070	0.011 – 0.129		2.311	**0.021**
Surprise*Autistic traits	0.033	-0.021 – 0.088		1.194	0.233
Neutral*Autistic traits†	0.041	-0.014 – 0.096		1.454	0.146
Emotion Category*Confidence*Autistic traits			1.702		0.131
Anger*Confidence*Autistic traits	-0.026	-0.080 – 0.028		-0.939	0.348
Fear*Confidence*Autistic traits	0.004	-0.050 – 0.058		0.144	0.885
Happiness*Confidence*Autistic traits	-0.008	-0.066 – 0.050		-0.263	0.793
Sadness*Confidence*Autistic traits	0.062	0.007 – 0.116		2.214	**0.027**

Surprise*Confidence*Autistic traits	0.023	-0.037 – 0.082	0.748	0.455
Neutral*Confidence*Autistic traits†	-0.055	-0.110 - -0.001	-1.932	0.053
**Random Effects**				
σ^2	0.49			
$\tau_{00\ ID}$	0.03			
$\tau_{00\ StimIdentity}$	0.04			
ICC	0.12			
N_{ID}	56			
$N_{StimIdentity}$	10			
Observations	3360			
Marginal R^2 / Conditional R^2	0.443 / 0.513			

†taken from general linear hypothesis calculation, z-score instead of t-value

Table S24. Results of the linear mixed-effects model predicting relative recognition accuracy of angry facial expressions by social anxiety trait, corrugator muscle activity and the 2-way interaction between social anxiety traits and corrugator muscle activity

Predictors	β	*CI*	*F*	*t*	*p*
Intercept	-0.143	-0.520 – 0.233	0.695	-0.834	0.421
Social anxiety traits	0.054	-0.077 – 0.186	0.829	0.829	0.410
Corrugator activity	-0.028	-0.124 – 0.068	0.304	-0.569	0.570
Corrugator activity* Social anxiety traits	0.088	-0.002 – 0.177	3.696	1.918	0.056
**Random Effects**					
σ^2	0.44				
$\tau_{00\ ID}$	0.19				
$\tau_{00\ StimIdentity}$	0.25				
ICC	0.50				
N_{ID}	57				
$N_{StimIdentity}$	10				
Observations	570				
Marginal R^2 / Conditional R^2	0.009 / 0.509				

Table S25. Results of the linear mixed-effects model predicting relative recognition accuracy of happy facial expressions by social anxiety traits, zygomaticus muscle activity, corrugator muscle activity and the 2-way interactions between social anxiety traits and each muscles activity

Predictors	β	*CI*	*F*	*t*	*p*
Intercept	0.647	0.409 – 0.885	35.749	5.978	**<0.001**
Zygomaticus activity	0.052	0.005 – 0.098	4.738	2.171	**0.030**
Social anxiety traits	-0.034	-0.112 – 0.044	0.644	-0.864	0.390
Corrugator activity	-0.017	-0.083 – 0.049	0.250	-0.499	0.618
Zygomaticus activity* Social anxiety traits	-0.014	-0.059 – 0.032	0.369	-0.587	0.558
Corrugator activity* Social anxiety traits	0.010	-0.057 – 0.077	0.118	0.298	0.766
**Random Effects**					
σ^2	0.17				
$\tau_{00\ ID}$	0.05				
$\tau_{00\ StimIdentity}$	0.10				
ICC	0.47				
N_{ID}	57				
$N_{StimIdentity}$	10				
Observations	570				
Marginal R^2 / Conditional R^2	0.012 / 0.481				

Table S26. Results of the linear mixed-effects model predicting relative recognition accuracy of fearful facial expressions by social anxiety traits, corrugator muscle activity and the 2-way interaction between social anxiety traits and corrugator muscle activity

Predictors	β	*CI*	*F*	*t*	*p*
Intercept	-0.826	-1.096 – -0.566	42.000	-6.481	**<0.001**
Social anxiety traits	0.001	-0.145 – 0.147	0.000	0.016	0.987
Corrugator activity	-0.051	-0.175 – 0.072	0.672	-0.819	0.413
Corrugator activity* Social anxiety traits	-0.043	-0.166 – 0.080	0.470	-0.686	0.493
**Random Effects**					
σ^2	0.50				
$\tau_{00\ ID}$	0.25				
$\tau_{00\ StimIdentity}$	0.11				

ICC	0.42
N $_{ID}$	57
N $_{StimIdentity}$	10
Observations	570
Marginal R^2 / Conditional R^2	0.003 / 0.417

Table S27. Results of the linear mixed-effects model predicting relative recognition accuracy of sad facial expressions by social anxiety traits, corrugator muscle activity and the 2-way interaction between social anxiety traits and corrugator muscle activity

Predictors	β	*CI*	*F*	*t*	*p*
Intercept	-0.616	-1.001 – -0.230	12.345	-3.514	**<0.001**
Social anxiety traits	0.030	-0.098 – 0.157	0.217	0.465	0.643
Corrugator activity	0.046	-0.082 – 0.174	0.492	0.702	0.483
Corrugator activity* Social anxiety traits	-0.003	-0.126 – 0.121	0.002	-0.046	0.963
**Random Effects**					
σ^2	0.59				
$\tau_{00\ ID}$	0.17				
$\tau_{00\ StimIdentity}$	0.27				
ICC	0.42				
N $_{ID}$	57				
N $_{StimIdentity}$	10				
Observations	570				
Marginal R^2 / Conditional R^2	0.001 / 0.426				

Table S28. Results of the linear mixed-effects model predicting relative recognition accuracy of angry facial expressions by autistic traits, corrugator muscle activity and the 2-way interaction between autistic traits and corrugator muscle activity

Predictors	β	*CI*	*F*	*t*	*p*
Intercept	-0.143	-0.521 – 0.235	0.689	-0.830	0.423
Autistic traits	-0.066	-0.199 – 0.066	0.999	-1.000	0.322
Corrugator activity	-0.011	-0.110 – 0.087	0.051	-0.225	0.822
Corrugator activity* Autistic traits	-0.057	-0.164 – 0.050	1.093	-1.045	0.296

Random Effects

σ^2	0.44
$\tau_{00\ ID}$	0.19
$\tau_{00\ StimIdentity}$	0.25
ICC	0.50
N_{ID}	56
$N_{StimIdentity}$	10
Observations	560
Marginal R^2 / Conditional R^2	0.008 / 0.508

Table S29. Results of the linear mixed-effects model predicting relative recognition accuracy of happy facial expressions by autistic traits, zygomaticus muscle activity, corrugator muscle activity and the 2-way interactions between autistic traits and each muscles activity

Predictors	β	*CI*	*F*	*t*	*p*
Intercept	0.651	0.414 – 0.889	36.299	6.025	**<0.001**
Zygomaticus activity	0.044	-0.002 – 0.090	3.603	1.898	0.058
Autistic traits	-0.065	-0.140 – 0.011	2.896	-1.702	0.093
Corrugator activity	-0.006	-0.073 – 0.061	0.030	-0.172	0.863
Zygomaticus activity* Autistic traits	0.008	-0.048 – 0.065	0.083	0.288	0.774
Corrugator activity* Autistic traits	0.049	-0.016 – 0.114	2.180	1.476	0.140
**Random Effects**					
σ^2	0.17				
$\tau_{00\ ID}$	0.05				
$\tau_{00\ StimIdentity}$	0.10				
ICC	0.47				
N_{ID}	56				
$N_{StimIdentity}$	10				
Observations	560				
Marginal R^2 / Conditional R^2	0.029 / 0.484				

Table S30. Results of the linear mixed-effects model predicting relative recognition accuracy of fearful facial expressions by autistic traits, corrugator muscle activity and the 2-way interaction between autistic traits and corrugator muscle activity

Predictors	β	*CI*	*F*	*t*	*p*
Intercept	-0.825	-1.087 – -0.564	44.699	-6.686	**<0.001**
Autistic traits	-0.171	-0.312 – -0.030	5.929	-2.435	**0.018**
Corrugator activity	-0.043	-0.165 – 0.080	0.463	-0.681	0.496
Corrugator activity* Autistic traits	-0.015	-0.137 – 0.106	0.061	0.247	0.805
**Random Effects**					
σ^2	0.50				
$\tau_{00\ ID}$	0.22				
$\tau_{00\ StimIdentity}$	0.10				
ICC	0.40				
N_{ID}	56				
$N_{StimIdentity}$	10				
Observations	570				
Marginal R^2 / Conditional R^2	0.035 / 0.416				

Table S31. Results of the linear mixed-effects model predicting relative recognition accuracy of sad facial expressions by autistic traits, corrugator muscle activity and the 2-way interaction between autistic traits and corrugator muscle activity

Predictors	β	*CI*	*F*	*t*	*P*
Intercept	-0.609	-0.997 – -0.222	11.979	-3.461	**<0.001**
Autistic traits	0.049	-0.081 – 0.178	0.566	0.752	0.455
Corrugator activity	0.048	-0.080 – 0.176	0.545	0.738	0.461
Corrugator activity* Autistic traits	-0.135	-0.256 – -0.013	4.757	-2.181	**0.030**
**Random Effects**					
σ^2	0.59				
$\tau_{00\ ID}$	0.17				
$\tau_{00\ StimIdentity}$	0.27				
ICC	0.43				
N_{ID}	56				
$N_{StimIdentity}$	10				

Observations	560
Marginal R^2 / Conditional R^2	0.007 / 0.430

Analysis on perceived emotional intensity

Data analysis

In order to explore whether social anxiety traits were associated with alterations in how emotionally intense the expressions were perceived, we calculated a LMM on perceived emotional intensity with emotion category, the trait dimension and their interaction as predictors. The identity of the stimulus and the participant ID were both added as random effects (random intercept), thus mirroring the other behavioural models. Coefficients for the emotion categories (main effects and interactions) were calculated by contrasting the respective category against the overall effect. For the neutral category, coefficients were calculated and tested (z-tests) using general hypotheses testing.

Results

Descriptive statistics. The six emotion categories also varied in how emotionally intense they were perceived, with highest ratings obtained for displays of happiness (M = 86.82, SD = 19.41), then neutral (M = 76.04, SD = 25.82), then surprise (M = 73.61, SD = 23.33), then fear (M = 64.56, SD = 25.53), then anger (M = 64.02, SD = 28.73) and lastly sadness (M = 62.00, SD = 28.29).

Social anxiety traits. In the first LMM on perceived emotional intensity, including emotion category, social anxiety traits and their interaction as predictors, perceived emotional intensity was significantly predicted by emotion category, $F(5,3344) = 106.141$, $p < .001$ and social anxiety traits, $F(1,55) = 5.365$, $p = .024$. Compared to the average perceived emotional intensity ratings, happy, neutral and surprised expressions were rated significantly higher in emotional intensity, happy: $\beta = 0.584$, $t(3344) = 18.542$, $p < .001$, neutral: $\beta = 0.181$, $z = 5.758$, $p < .001$, surprised: $\beta = 0.091$, $t(3344) = 2.889$, $p = .004$. Sad, angry and fearful expressions, on the other hand, were perceived as significantly less emotionally intense than average, sad: $\beta = -0.342$, $t(3344) = -10.872$, $p < .001$, angry: $\beta = -0.267$, $t(3344) = -8.481$, $p < .001$, fearful: $\beta =-0.247$, $t(3344) = -7.837$, $p < .001$. Contrary to our expectations, social anxiety traits were negatively related to emotional intensity judgments $\beta = -0.129$, $t(55) = -2.316$, $p = 0.024$, Further, we did not find a significant interaction between emotion category and social anxiety traits. Thus, in line with the accuracy results, we did not find support for a heightened sensitivity to specifically negative facial expressions with higher social anxiety traits in the perceived emotional intensity ratings (see Fig. S3(A) and Table S32).

Autistic traits. Both emotion category, $F(5,3285) = 105.870$, $p < .001$, and the interaction between emotion category and autistic traits, $F(5, 3285) =11.213$, $p < .001$, were significant predictors in the second LMM on perceived emotional intensity. While facial displays of sadness and fear were experienced as more intense with higher autistic traits, $\beta = 0.149$, $t(3285) = 4.650$, $p < .001$, and $\beta = 0.097$, $t(3285) = 3.023$, $p = .003$ respectively, the opposite applied to happy and neutral expressions. Both categories were rated less emotionally intense with higher autistic traits, $\beta = -0.134$, $t(3285) = -4.197$, $p < .001$, and $\beta = -0.135$, $z = -4.227$, $p < .001$ respectively (see Fig. S3 (B) and Table S33). Taken together, the relationship between autistic traits and perceived emotional intensity seems to depend on the displayed expression.

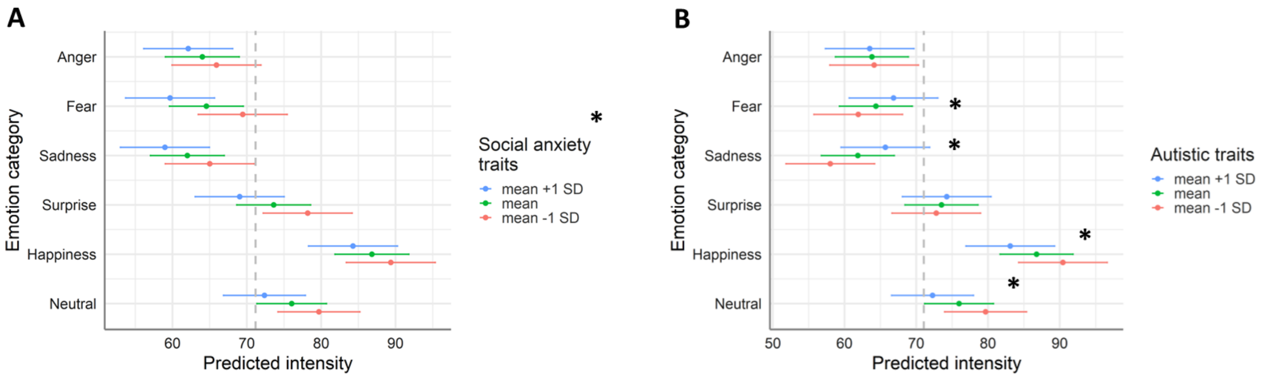

Fig. S3. Predicted perceived intensity ratings depending on (A) social anxiety trait levels and (B) autistic trait levels by emotion category (anger, fear, sadness, surprise happiness, neutral). For illustrative purposes, predicted accuracies for mean values as well as mean values +/- 1 SD of the continuous variables social anxiety traits and autistic traits are depicted. Whiskers represent confidence intervals and significant effects are marked with an asterisk.

Short discussion

Interestingly, the expressions' perceived emotional intensity ratings mirrored the overall pattern of the confidence ratings (see Results section in manuscript), with happy, surprised and neutral expressions receiving ratings above, and angry, fearful and sad receiving ratings below the average. This overlap might be explained by differences in facial expressiveness between the stimuli which was shown to influence both estimated readability and actual readability of expressions (Alkhaldi et al., unpublished results). Hence, less expressive displays might have been rated as both less intense and more difficult to classify (lower confidence).

Model fits

Table S32. Results of the linear mixed-effects model predicting perceived emotional intensity by emotion category, social anxiety traits and their interaction

Predictors	β	*CI*	*F*	*t*	*p*
Intercept	0.000	-0.185 – 0.185	0.0000	0.000	1.000
Emotion Category			106.141		**<0.001**
Anger	-0.267	-0.329 – -0.205		-8.481	**<0.001**

Fear	-0.247	-0.308 – -0.185		-7.837	**<0.001**
Happiness	0.584	0.522 – 0.645		18.542	**<0.001**
Sadness	-0.342	-0.404 – -0.280		-10.872	**<0.001**
Surprise	0.091	0.029 – 0.153		2.889	**0.004**
Neutral†	0.181	0.120 - 0.243		5.758	**<0.001**
Social anxiety traits	-0.129	-0.241 – -0.017	5.365	-2.316	**0.024**
Emotion Category*Social anxiety traits			1.593		0.159
Anger*Social anxiety traits	0.058	-0.005 – 0.120		1.818	0.069
Fear*Social anxiety traits	-0.055	-0.117 – 0.007		-1.728	0.084
Happiness*Social anxiety traits	0.033	-0.029 – 0.096		1.051	0.293
Sadness*Social anxiety traits	0.016	-0.047 – 0.078		0.493	0.622
Surprise* Social anxiety traits	-0.043	-0.105 – 0.019		-1.357	0.175
Neutral*Social anxiety traits†	-0.009	-0.071- 0.053		-0.276	0.782

Random Effects

σ^2	0.68
$\tau_{00\ ID}$	0.16
$\tau_{00\ StimIdentity}$	0.05
ICC	0.24
$N_{StimIdentity}$	10
N_{ID}	57
Observations	3420
Marginal R^2 / Conditional R^2	0.122 / 0.331

†taken from general linear hypothesis calculation, z-score instead of t-value

Table S33. Results of the linear mixed-effects model predicting perceived emotional intensity by emotion category, autistic traits and their interaction

Predictors	β	*CI*	*F*	*t*	*p*
Intercept	-0.005	-0.194 – 0.185	0.003	-0.051	0.960
Emotion Category			105.870		**<0.001**

Anger	-0.270	-0.332 – -0.207		-8.508	**<0.001**
Fear	-0.249	-0.311 – -0.187		-7.857	**<0.001**
Happiness	0.586	0.524 – 0.648		18.498	**<0.001**
Sadness	-0.343	-0.405 – -0.281		-10.820	**<0.001**
Surprise	0.092	0.030 – 0.154		2.904	**0.004**
Neutral†	0.183	0.121 - 0.245		5.783	**<0.001**
Autistic traits	-0.004	-0.123 – 0.115	0.005	-0.069	0.946
Emotion Category*Autistic traits			11.213		**<0.001**
Anger*Autistic traits	-0.008	-0.070 – 0.055		-0.238	0.812
Fear*Autistic traits	0.097	0.034 – 0.159		3.023	**0.003**
Happiness*Autistic traits	-0.134	-0.197 – -0.072		-4.197	**<0.001**
Sadness*Autistic traits	0.149	0.086 – 0.211		4.650	**<0.001**
Surprise*Autistic traits	0.032	-0.031 – 0.094		0.988	0.323
Neutral*Autistic traits†	-0.135	-0.198 - -0.072		-4.227	**<0.001**

Random Effects

σ^2	0.67
$\tau_{00\ ID}$	0.18
$\tau_{00\ StimIdentity}$	0.05
ICC	0.26
$N_{StimIdentity}$	10
N_{ID}	56
Observations	3360
Marginal R^2 / Conditional R^2	0.115 / 0.341

†taken from general linear hypothesis calculation, z-score instead of t-value
